# CWH43 Is a Novel Tumor Suppressor Gene with Negative Regulation of TTK in Colorectal Cancer

**DOI:** 10.3390/ijms242015262

**Published:** 2023-10-17

**Authors:** Cheng-Chin Lee, Kuei-Yen Tsai, Ai-Wei Lee, Po-Li Wei, Chien-Yu Huang, Uyanga Batzorig, Yu-Jia Chang

**Affiliations:** 1 Graduate Institute of Medical Sciences, College of Medicine, Taipei Medical University, Taipei 11031, Taiwan; kerwinpipi@gmail.com; 2 Graduate Institute of Clinical Medicine, College of Medicine, Taipei Medical University, Taipei 11031, Taiwan; leiftsai@gmail.com; 3 Department of Surgery, School of Medicine, College of Medicine, Taipei Medical University, Taipei 11031, Taiwan; poliwei@tmu.edu.tw; 4 Division of General Surgery, Department of Surgery, Shuang Ho Hospital, Taipei Medical University, New Taipei City 23561, Taiwan; 5 Department of Anatomy and Cell Biology, School of Medicine, College of Medicine, Taipei Medical University, Taipei 11031, Taiwan; 6 Division of Colorectal Surgery, Department of Surgery, Taipei Medical University Hospital, Taipei Medical University, Taipei 11031, Taiwan; 7 Cancer Research Center and Translational Laboratory, Taipei Medical University Hospital, Taipei Medical University, Taipei 11031, Taiwan; 8 Graduate Institute of Cancer Biology and Drug Discovery, Taipei Medical University, Taipei 11031, Taiwan; 9 School of Medicine, National Tsing Hua University, Hsinchu 30013, Taiwan; cyhuang@life.nthu.edu.tw; 10 Institute of Molecular and Cellular Biology, National Tsing Hua University, Hsinchu 30013, Taiwan; 11 Department of Dermatology, University of California, San Diego, La Jolla, CA 92093, USA; 12 Cell Physiology and Molecular Image Research Center, Wan Fang Hospital, Taipei Medical University, Taipei 11031, Taiwan; 13 Department of Pathology, Wan Fang Hospital, Taipei Medical University, Taipei 11031, Taiwan

**Keywords:** CWH43, CRC, TTK, proliferation, migration, biomarker

## Abstract

Colorectal cancer (CRC) ranks among the most prevalent forms of cancer globally, and its late-stage survival outcomes are less than optimal. A more nuanced understanding of the underlying mechanisms behind CRC’s development is crucial for enhancing patient survival rates. Existing research suggests that the expression of Cell Wall Biogenesis 43 C-Terminal Homolog (*CWH43*) is reduced in CRC. However, the specific role that CWH43 plays in cancer progression remains ambiguous. Our research seeks to elucidate the influence of CWH43 on CRC’s biological behavior and to shed light on its potential as a therapeutic target in CRC management. Utilizing publicly available databases, we examined the expression levels of CWH43 in CRC tissue samples and their adjacent non-cancerous tissues. Our findings indicated lower levels of both mRNA and protein expressions of CWH43 in cancerous tissues. Moreover, we found that a decrease in *CWH43* expression correlates with poorer prognoses for CRC patients. In vitro experiments demonstrated that the suppression of CWH43 led to increased cell proliferation, migration, and invasiveness, while its overexpression had inhibitory effects. Further evidence from xenograft models showed enhanced tumor growth upon CWH43 silencing. Leveraging data from The Cancer Genome Atlas (TCGA), our Gene Set Enrichment Analysis (GSEA) indicated a positive relationship between low *CWH43* expression and the activation of the epithelial–mesenchymal Transition (EMT) pathway. We conducted RNA sequencing to analyze gene expression changes under both silenced and overexpressed CWH43 conditions. By identifying core genes and executing KEGG pathway analysis, we discovered that CWH43 appears to have regulatory influence over the TTK-mediated cell cycle. Importantly, inhibition of TTK counteracted the tumor-promoting effects caused by CWH43 downregulation. Our findings propose that the decreased expression of CWH43 amplifies TTK-mediated cell cycle activities, thus encouraging tumor growth. This newly identified mechanism offers promising avenues for targeted CRC treatment strategies.

## 1. Introduction

Colorectal cancer (CRC) is the third most diagnosed cancer globally and stands as the second primary cause of cancer-associated fatalities [1]. As of 2020, CRC was responsible for 10% of all new cancer cases and accounted for 9.4% of cancer-associated deaths [2]. The onset of this disease is influenced by a multitude of factors, spanning genetic predispositions, environmental triggers, and lifestyle choices [3]. Although strides have been made in multidisciplinary treatments, leading to enhanced patient survival rates, the five-year overall survival statistic for cases of metastatic CRC remains a disheartening 10.5% [4]. Contemporary treatment approaches for advanced-stage CRC encompass chemotherapy, targeted therapeutic interventions, and immunotherapy [5]. Nonetheless, the persistent issue of drug resistance serves as a significant barrier to successful treatment, often resulting in tumor relapse and metastasis. Hence, there is an urgent need for reliable markers for early detection and surveillance of disease progression in CRC, along with a more comprehensive understanding of the mechanisms propelling tumor growth to devise effective treatment options.

Glycosylphosphatidylinositol (GPI) is a type of glycerophospholipid that functions as a lipid anchor, attaching to the C-terminus of proteins and facilitating their delivery to the external side of the plasma membrane. The foundational structure of GPI consists of inositol phospholipids, glycans made up of one glucosamine and three mannose units, finished with an ethanolamine phosphate (EtNP) [6]. GPI anchoring plays a pivotal role in processes like mammalian embryogenesis, development, neurogenesis, fertilization, and immune response [7]. The protein-coding gene Cell Wall Biogenesis 43 C-Terminal Homolog (CWH43) encodes for the PGAP2 (post-GPI attachment to proteins 2)-interacting protein and is believed to participate in GPI anchor synthesis [8]. In the yeast Saccharomyces cerevisiae, the N-terminal portion of CWH43 showcases a sequence that is analogous to the mammalian PGAP2, which is instrumental in converting the lipid part of GPI anchors to ceramides [9,10]. In humans, mutations impacting the remodeling of the GPI lipid component have been linked to hereditary spastic paraplegias, a group of neurodegenerative motor neuron conditions, identified through exome sequencing [11].

Phosphotyrosine-Picked Threonine-Protein Kinase (TTK), also recognized as Mps1, constitutes an essential component of the spindle assembly checkpoint (SAC), ensuring the precise segregation of chromosomes to daughter cells during cell division [12]. TTK dysregulation has been closely associated with aneuploidy, chromosomal anomalies, and tumorigenesis [13]. Heightened TTK expression has been consistently documented across diverse cancer types, encompassing lung [14], breast [15], liver [16], kidney [17], colon [18], and gastric cancer [19]. Comprehending the multifaceted roles of TTK in cancer is imperative for the development of targeted therapeutic strategies and the enhancement of patient prognosis [20].

While the precise role of CWH43 in humans remains to be elucidated, its presence is notably enriched within the epithelial layers of the gastrointestinal system, including the stomach, colon, and rectum [21,22]. Prior research has highlighted a reduction in *CWH43* expression in cases of CRC [23]. Another investigative endeavor utilized microarray gene expression profiles to create a predictor classifier. This classifier identified a declining trend in *CWH43* expression from normal mucosa to adenoma, then to carcinoma, positioning *CWH43* as a potential early-stage biomarker for CRC [24]. Our study unearthed diminished levels of *CWH43* expression in CRC tumor samples, sourced from public databases. Notably, these expression levels exhibited correlations with clinical outcomes for CRC patients. Both in vitro and in vivo assessments have illuminated CWH43’s involvement in cell proliferation and invasion processes. Digging deeper, we found links between CWH43 functions, the epithelial–mesenchymal transition (EMT), and cell cycle regulation. Intervening with an inhibitor targeting a cell-cycle-related gene mitigated the impacts of CWH43 knockdown on CRC cell viability and migratory tendencies. This research underscores the potential of CWH43 not only as a diagnostic and monitoring tool but also as a focal point in pioneering new therapeutic approaches.

## 2. Results

### 2.1. CWH43 in CRC and Its Implications for Patient Outcomes

**Figure 1:**
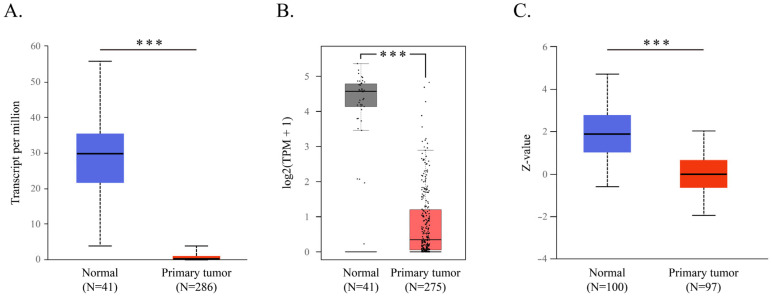
Comparison of CWH43 expression in normal and CRC samples. Analysis of CWH43 mRNA expression in colon adenocarcinoma (COAD) and standard tissues from (**A**) UALCAN and (**B**) GEPIA resources (Dots represent jittered points). (**C**) Protein levels of CWH43 in colon cancer as seen via UALCAN, sourced from the Clinical Proteomic Tumor Analysis Consortium database. The Z-value represents the standard deviation from the median across samples. *** *p* < 0.001.

**Figure 2:**
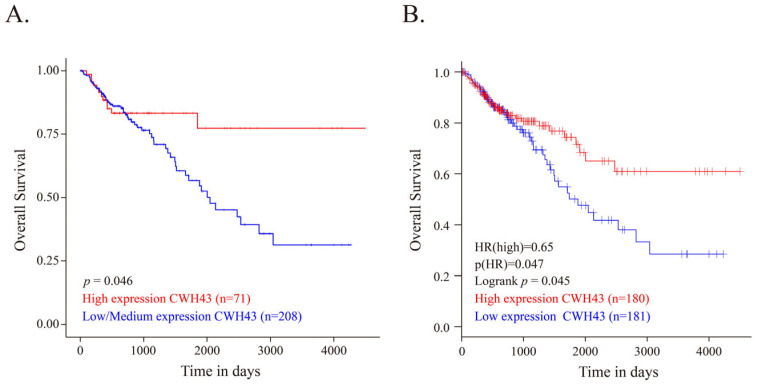
Correlation between *CWH43* expression levels and survival in CRC patients. Kaplan–Meier survival plots depicting overall survival of CRC patients based on high versus low *CWH43* expression from the (**A**) UALCAN (specifically for colon cancer) and (**B**) GEPIA resources (encompassing both colon and rectal cancer).

In assessing the connection between CWH43 expression and CRC, the UALCAN and GEPIA databases revealed decreased CWH43 mRNA expression in CRC tissues compared to normal ones (Figure 1A,B). A corresponding reduction in CWH43 protein was also evident in primary CRC tumors (Figure 1C). Significantly, patients with higher *CWH43* expression demonstrated better overall survival rates than their low/medium expression counterparts (Figure 2A,B). These data indicate that lower *CWH43* expression in CRC correlates with suboptimal long-term survival, suggesting its critical role in CRC pathogenesis.

### 2.2. CWH43’s Influence on CRC Tumorigenesis and Cell Growth

**Figure 3:**
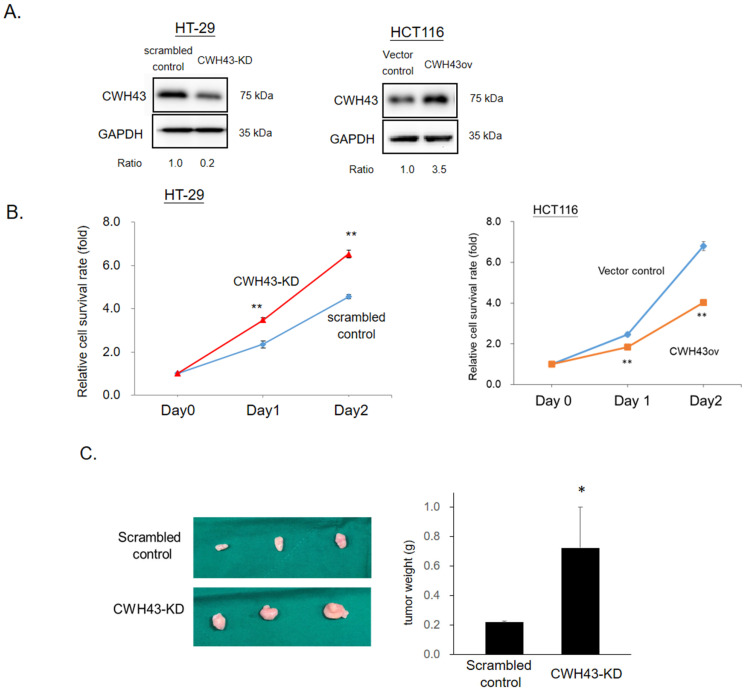
Impact of CWH43 on CRC cell growth. Protein levels of CWH43 in (**A**) upregulated HT29 cells and downregulated HCT116 cells. (**B**) The SRB assay was utilized to gauge the relative survival rates of the cells. (**C**) Xenograft model showed tumor size and weight of scrambled control and CWH43-KD groups. All experiments were conducted in triplicate, and all data were expressed as mean + SD. Two-tailed Student’s *t*-tests were used to assess statistical significance. * *p* < 0.05, ** *p* < 0.005.

The CellExpress database (GSE36133) indicated that *CWH43* expression is generally lower in most CRC cell lines (Figure S1). To further validate CWH43’s influence on cancer cell proliferation, we engineered HT-29 cells with reduced CWH43 levels (CWH43-KD) and HCT116 cells with elevated CWH43 expression (CWH43ov) (See Figure 3A). Western blotting was used to confirm the changes in CWH43 levels. As illustrated in Figure 3B, growth activity was found to be elevated in CWH43-KD cells compared to their scrambled controls. Conversely, enhanced expression of CWH43 in HCT116 cells led to a decline in growth activity. In this context, our findings in both HT-29 and HCT116 cell lines underscore an inverse correlation between CWH43 expression and the rate of CRC cell proliferation (Refer to Figure 3B). In a xenograft experiment, DLD-1 cells with reduced CWH43 (CWH43-KD) showed quicker growth than their control counterparts (see Figure 3C).

### 2.3. Involvement of CWH43 in CRC Migration, Invasion, and EMT Regulation

**Figure 4:**
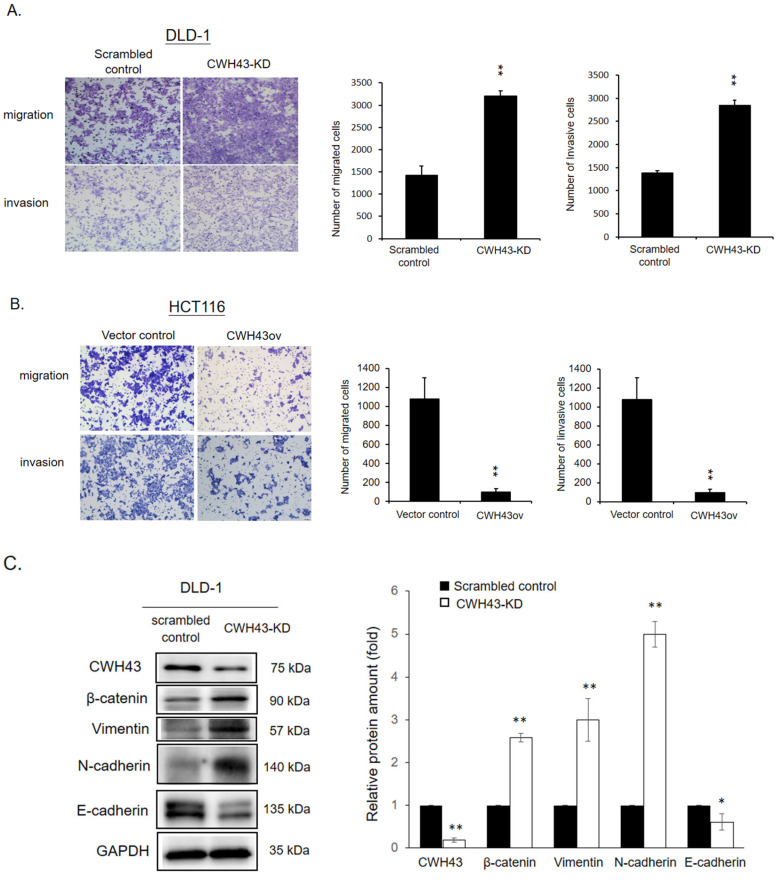
Role of CWH43 in restraining CRC cell metastasis. Evaluating the migration and invasion capacities of standard versus CWH43-altered cells in (**A**) HCT116 and (**B**) DLD-1 samples (100× magnification). (**C**) Western blot analysis was used to determine expression levels of beta-catenin, vimentin, N-cadherin, and E-cadherin in CWH43-suppressed DLD-1 cells. Statistical significance was evaluated using two-tailed Student’s *t*-tests on all experiments conducted in triplicate. * *p* < 0.05, ** *p* < 0.005.

Our tests showcased CWH43’s influence on the migration and invasion capabilities of CRC cells. Knockdown of CWH43 increased migration and invasion in DLD-1 cells, whereas its overexpression inhibited these traits in HCT116 cells (Figure 4A,B). Subsequent GSEA analysis of the TCGA dataset highlighted *CWH43*’s negative association with several signaling pathways, most notably the epithelial–mesenchymal Transition (Figure S2A–C). Furthermore, CWH43’s impact on the expression of key proteins like E-cadherin, β-catenin, Vimentin, and N-cadherin underpins its role in EMT regulation (Figure 4C).

### 2.4. CWH43’s Regulatory Impact on Threonine Tyrosine Kinase (TTK) in CRC

**Figure 5:**
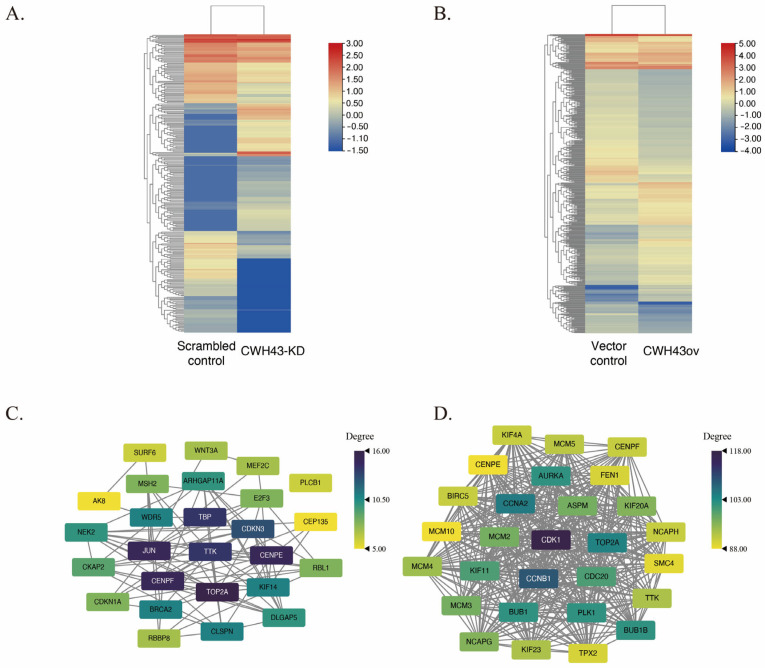
Identification of DEGs and core genes via RNA-seq. A heatmap depicts the grouping of differentially expressed genes (criteria: *p* adj < 0.05, |Log2 fold change| ≥ 1) between standard and (**A**) CWH43-downregulated (251 DEGs) or (**B**) CWH43-upregulated cells (415 DEGs). Protein interactions among core genes were extracted from the DEGs of (**C**) CWH43-downregulated and (**D**) CWH43-upregulated cells using the CytoNCA plugin in Cytoscape.

To explore the enigmatic function of CWH43 in the development of cancer, we delved into its downstream regulatory mechanisms and linked genes in CRC. We employed RNA sequencing to identify potential target genes affected by CWH43 and hence, to gain insights into how it impacts the progression of CRC. Comparing CWH43-KD and overexpressed CWH43 HCT116 cells to a control group revealed differentially expressed genes (DEGs) with a log2 fold change of 1 or greater (as shown in Figure 5A,B). We found 251 DEGs in the CWH43-KD cells (Table S1) and 415 in the CWH43-overexpressing cells (Table S2). To pinpoint central genes among the DEGs, we used the STRING database to build a protein–protein interaction (PPI) network. This network was then analyzed using the Cytoscape software (Figure 5C,D). Our analysis identified 27 central genes in CWH43-KD cells (Table S3) and 28 in CWH43-overexpressing cells (Table S4). We found TTK was shown in the central genes in CWH43-KD and CWH43ov cells.

### 2.5. KEGG Pathway Analysis and Its Regulatory Influence on TTK Expression in the Cell Cycle

**Figure 6:**
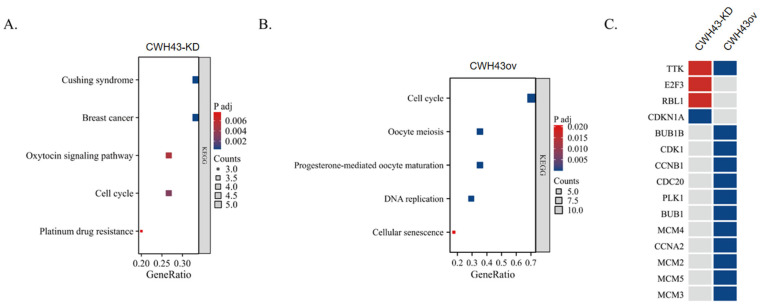
KEGG pathways influenced by core genes. A bubble chart visualizes KEGG pathways for (**A**) CWH43-KD and (**B**) CWH43ov cells. (**C**) The hsa04110 pathway (pertaining to the cell cycle) emerged as a consistently influenced pathway. Red signifies upregulated genes, blue denotes downregulated genes, while grey represents no notable change.

Next, KEGG pathway analysis was carried out to determine the likely roles of these central genes in CRC. In CWH43-KD cells, the most enriched pathways included Cushing syndrome, Breast cancer, Oxytocin signaling, Cell cycle, and Resistance to platinum drugs (Figure 6A). For CWH43-overexpressing cells, the top pathways were Cell cycle, Oocyte meiosis, Progesterone-mediated oocyte maturation, DNA replication, and Cellular senescence (Figure 6B).

Notably, the “Cell cycle” pathway appeared as a common enriched pathway for both CWH43 knockdown and overexpression groups. Further scrutiny of genes related to the cell cycle pathway showed that TTK expression increased in CWH43-KD cells but decreased in CWH43-overexpressing cells (Figure 6C). In summary, our data suggest that CWH43 is involved in the cell cycle and may serve as a regulatory factor for TTK expression in CRC.

### 2.6. TTK Inhibitor Reversed Tumor-Promoting Effect in CWH43-KD Cancer Cells

**Figure 7:**
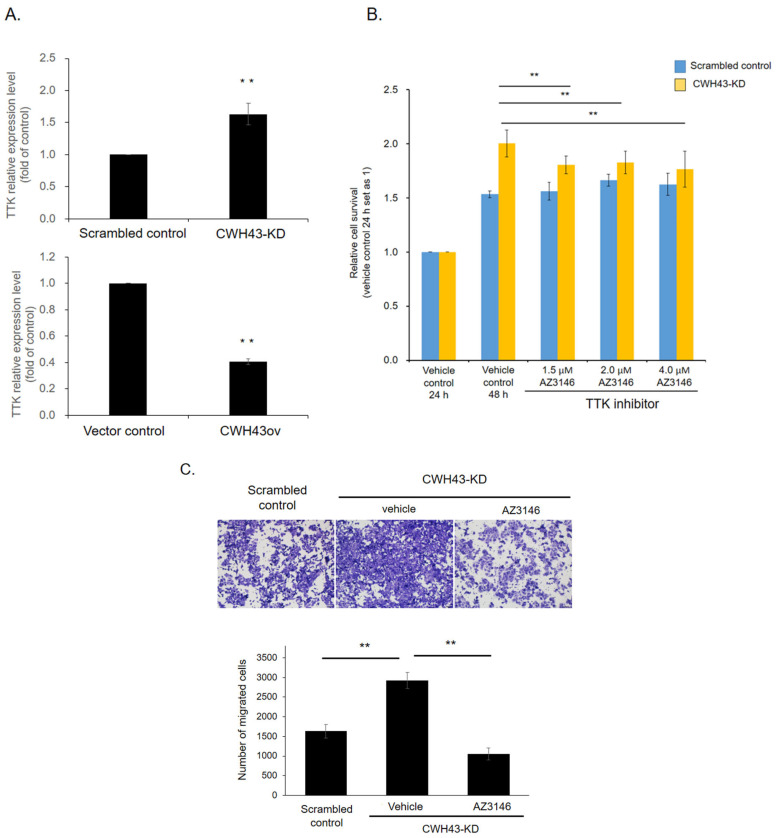
Counteractive role of TTK on CWH43-manipulated cells. (**A**) Quantitative PCR (qPCR) assessed TTK expression in cells with CWH43-KD and CWH43ov cells. (**B**) The TTK inhibitor diminished growth in the CWH43-KD cells but left the scrambled control group unaffected. (**C**) The TTK inhibitor markedly curtailed migration in CWH43-KD cells (100× magnification). The experiments were conducted independently in triplicate. Statistical significance was determined by a two-tailed Student’s *t*-test. ** represent *p* < 0.005, respectively.

To determine if the tumor-promoting effects of CWH43-KD on CRC operate through TTK, we employed RT-qPCR assay. It was evident that *TTK* expression was markedly upregulated in CWH43-KD cells when contrasted with control cells (Figure 7A). Conversely, in HCT 116 cells where CWH43 was overexpressed, TTK mRNA levels declined. This suggests that CWH43 could function by negatively regulating TTK. To further investigate this mechanism, we introduced a TTK inhibitor, AZ3146. Our findings, illustrated in Figure 7B, show that introducing AZ3146 to CWH43-KD cells notably reduced the relative cell survival rate when contrasted with the vehicle control. However, the TTK inhibitor did not influence cell survival in the scrambled control cells. In line with this, the transwell migration activity also decreased post-AZ3146 treatment in CWH43-KD cells (Figure 7C). These observations suggest that CWH43’s tumor-suppressing capability might operate by negatively impacting TTK.

## 3. Discussion

In humans, previous studies have associated CWH43 with normal pressure hydrocephalus [25,26]. Yet, its role in cancer remains undefined. Our research uncovers its significant function in the development of colorectal cancer. Initially, we observed a marked reduction in *CWH43* expression in CRC tissues, linking its low levels to an adverse survival outcome in CRC patients (Figure 1 and Figure 2). Furthermore, suppressing CWH43 amplified CRC cell growth and tumor expansion in mice, whereas its increased expression curtailed CRC cell viability (Figure 3). Additionally, reduced CWH43 heightened CRC cell migration, invasion, and epithelial–mesenchymal transition (Figure 4). These effects appeared to stem from TTK regulation (Figure 5 and Figure 6). Importantly, inhibiting TTK reversed these detrimental cellular behaviors in CWH43-suppressed CRC cells. This suggests that CWH43’s tumor-suppressing potential might act via TTK modulation. Given the persistent challenges in treating metastatic CRC and drug resistance, there is an urgent need for early-detection biomarkers. Our findings propose CWH43’s pivotal role in CRC development, presenting it as a potential preventive measure and therapeutic target.

The epithelial–mesenchymal transition (EMT) is a developmental program allowing stationary epithelial cells to acquire migratory and invasive capabilities. Tumor cells often exploit EMT to undergo molecular changes, transitioning partially from an epithelial to a mesenchymal phenotype [27]. EMT is intricately linked to numerous malignant traits in tumor cells, encompassing migration, invasion, stemness, and chemo-radiotherapy resistance [28]. Despite its pivotal role in tumor metastasis and sustaining hallmark features, the EMT signaling network remains incompletely understood, presenting challenges for potential clinical trials targeting EMT in cancer therapy [29]. We noted augmented migratory and invasive capabilities in CWH43 knockdown cells concomitant with the upregulation of epithelial–mesenchymal transition (EMT) markers. This implies that the tumor-associated downregulation of CWH43 may instigate cancer cells towards EMT, consequently fostering metastasis. Additional investigations are merited to elucidate the intricate regulatory mechanisms governing the crosstalk between CWH43 and EMT. These insights hold promise for advancing the development of targeted therapies to inhibit EMT.

In yeast, CWH43 is crucial for lipid transformations to ceramides, facilitating ceramide integration into GPI-anchored protein [30]. A lack of CWH43 disrupts GPI expression on yeast cell walls that is vital for their growth and survival [31]. Similarly, in human cells, CWH43 governs GPI-anchored protein targeting [25]. Yet, its function concerning cancer remains elusive. One meta-analysis spotlighted *CWH43* as one of the key genes displaying differential expression between CRC and standard mucosa using cDNA microarrays [23]. Another pinpointed *CWH43* as a central gene in gastric cancer via weighted gene co-expression network analysis [32]. Aligning with these findings, our research corroborates CWH43’s tumor-suppressing role, backed by both in vitro and in vivo tests.

Chromosomal separation during cell division relies on the spindle assembly checkpoint. Here, TTK (also labeled as MPS1) emerges as a critical regulator [33,34]. TTK’s role in preserving genomic integrity is crucial, with its irregularities associated with various cancers such as breast, liver, and lung cancers [14,16,35,36,37]. For colon cancer, one investigation deduced that TTK expression is notably elevated, correlating with adverse patient prognosis and heightened cell proliferation [18]. Another asserted that increased TTK disrupts the spindle assembly checkpoint, fostering genome instability and tumor growth in colon cells [38]. Echoing these findings, our study posits that CWH43 acts by negatively influencing TTK, influencing cancer development, invasion, and long-term patient outcomes.

Though we have unveiled CWH43’s tumor-suppressing role in CRC and its potential interaction with TTK, the exact mechanisms remain elusive. TTK’s role might extend beyond spindle assembly checkpoint maintenance. For instance, one study found TTK expression peaking in stage II clinical CRC tissues rather than in later stages [39]. Another highlighted TTK’s unique regulatory role in tumor cell viability, mediated by its interaction with mitochondria. Other research found CRC with microsatellite instability (MSI) presented TTK frameshift mutations [40], with TTK expression heightened in MSI-high status cancers versus those MSI-low [18]. In recent years, an expanding corpus of research has spotlighted TTK as a promising target for cancer therapy [41,42]. The inhibition of TTK prompts a premature exit of cancer cells from mitosis, culminating in heightened chromosome segregation errors and the genesis of aneuploid cells. Through successive rounds of cell division, cumulative chromosome segregation errors can ultimately trigger apoptosis in cancer cells [43]. Consequently, TTK has garnered substantial attention as a pivotal focus in cancer research, with TTK inhibitors undergoing escalating evaluation in clinical trials [44]. This underscores the need to further investigate the intricate CWH43–TTK relationship in CRC development.

In summary, our research indicates that decreased CWH43 expression may contribute to CRC progression by activating TTK. This highlights the potential of CWH43 as a promising target for CRC treatment, warranting more in-depth studies.

## 4. Materials and Methods

### 4.1. Gene Expression Level, Protein Expression Level, and Patient Survival Related to CWH43 in Colorectal Cancer

The University of Alabama at Birmingham cancer data analysis portal (UALCAN) is an online interactive portal which enables easy exploring and analysis of gene expression, cancer proteomics, and patient survival data obtained from The Cancer Genome Atlas (TCGA) database [45] and the Clinical Proteomic Tumor Analysis Consortium (CPTAC) database. UALCAN is accessible at http://ualcan.path.uab.edu (accessed on 5 June 2023). In addition, Gene Expression Profiling Interactive Analysis (GEPIA) provides differential expression analysis of tumor versus normal tissue, as well as functions for analysis by cancer type or pathological stage and patient survival analysis, based on TCGA and The Genotype-Tissue Expression (GTEx) project [46]. GEPIA is available at http://gepia.cancer-pku.cn/ (accessed on 24 July 2023). These tools were used to identify the relationship between CWH43 and colorectal cancer.

We performed gene expression analysis on large intestine cell lines using the Cancer Cell Line Encyclopedia (CCLE) (GSE36133) dataset available on the CellExpress website (http://cellexpress.cgm.ntu.edu.tw, accessed on 5 June 2023) [47].

### 4.2. Functional Enrichment Analysis

The RNA-seq data for the TCGA COADREAD (colorectal adenocarcinoma) project, processed using the STAR workflow, were acquired from the TCGA database (https://portal.gdc.cancer.gov, accessed on 16 August 2022). We utilized the edgeR [v3.38.2] package to perform differential gene expression analysis between the high and low expression groups of CWH43 in TCGA COADREAD data [48]. Gene Set Enrichment Analysis (GSEA) and Kyoto Encyclopedia of Genes and Genomes (KEGG) were performed using the clusterProfiler [v4.4.4] package [49]. The generated results were then visualized using the ggplot2 [v3.3.6] package.

### 4.3. Chemicals, Reagents, and Cell Culture

Human CRC cells, DLD-1 (CCL-221), HT-29 (HTB-38), and HCT116 (CCL-247), were obtained from American Type Culture Collection (ATCC, Rockville, MD, USA). All cells were cultured in RPMI 1640 medium supplemented with 10% fetal bovine serum (FBS) (SAFC Biosciences, Lenexa, KS, USA) and 1% penicillin/streptomycin containing 100 IU/mL of penicillin and 100 μg/mL of streptomycin at 37 °C in 5% CO_2_ in a humidified incubator. TTK inhibitor AZ3146 (Selleck, Houston, TX, USA) was dissolved in DMSO at a concentration of 100 mM and diluted sequentially into 4 mM, 2 mM, and 1.5 mM with DMEM containing 10% FBS.

### 4.4. Transfection and Generation of Stable Colonies

To knock down CWH43 expression, short hairpin RNA (shRNA; TRCN0000417738 and TRCN0000429495) targeting human CWH43 (NM_025087) was obtained from the National RNAi Core Facility at Academia Sinica in Taiwan. CWH43-shRNA and nontarget shRNA were transfected into HCT 116, DLD-1, and HT-29 cells, and stably transfected cells were selected using puromycin for 2 weeks. The expression level of *CWH43* was determined through quantitative reverse transcription polymerase chain reaction (RT-qPCR). To overexpress CWH43, the pCMV6-Entry-CWH43 (CAT#: RC224386, OriGene Technologies, Inc., Rockville, MD, USA) was transfected into HCT 116 cells through electroporation. Stably transfected cells were selected after adding G418, and the cells were used for subsequent experiments after confirming CWH43 overexpression through RT-qPCR assay and Western blotting.

### 4.5. Examination of Cell Viability

The cytotoxicity assay sulforhodamine B (SRB) assay was used to examine cell viability. Cells at a density of 2 × 10^4^ with vector control, CWH43 knockdown, and CWH43 overexpressed cells were seeded into 24-well plates (Falcon, Munich, Germany) and incubated at 37 °C in a 5% CO_2_ humidified incubator attached overnight. After incubation for 48 h, the cells were fixed with 10% (wt/vol) trichloroacetic acid at 4 °C overnight and then stained with 0.4% *w*/*v* protein-bound SRB for 30 min at room temperature. The stained cells were washed twice with 1% acetic acid and air-dried overnight. The protein-bound dye was dissolved in 10 mM Tris base solution, and the optical density (OD) was measured at 515 nm using a microplate reader (Bio-Rad Laboratories, Hercules, CA, USA). The baseline was defined as cells treated with control, while fold changes were calculated as the OD values of CWH43 overexpressed or knockdown cells relative to the baseline.

### 4.6. Transwell Migration and Invasion Assay

The BD Falcon cell culture insert and BD BioCoat Matrigel invasion chambers precoated with BD Matrigel matrix (BD Biosciences, Franklin Lakes, NJ, USA) were used, respectively, for in vitro cell migration and invasion assays. We seeded aliquots of 1 × 10^5^ cells suspended in 500 μL of serum-free RPMI medium into the upper compartment of each chamber, and the lower compartments were filled with 1 mL of RPMI medium containing 10% fetal bovine serum and 1% penicillin and streptomycin. After incubation for 48 h at 37 °C in a 5% CO_2_ incubator, each well and chamber was washed once with 1 mL of 1× PBS. The cells were fixed in less than 1 mL of methyl alcohol solution for a few seconds. The cells in the top chamber (non-migrated) were mechanically removed with cotton swabs. The cells on the reverse side were stained with 0.1% crystal violet. After the plate was incubated at room temperature for 8 h, the crystal violet was removed, and the number of stained cells was counted using a microscope (Olympus IX; Olympus, Tokyo, Japan) at 10-fold magnification. The number of migrated cells was counted using a handheld cell counter.

### 4.7. Animal Model

CB17 severe combined immunodeficient (SCID) male mice were randomly divided into experimental and control groups (*n* = 6 per group). The mice were inoculated with 2.5 × 10^7^ DLD-1 cells resuspended in a 50% mixture of Matrigel (BD Biosciences) in HBSS (Life Technologies, Carlsbad, CA, USA) into the right flank subcutaneous tissue. Tumor dimensions and body weights were measured twice a week using the following formula: tumor volume (mm^3^) = length × (width^2^)/2. After 4 weeks, the mice were sacrificed and the nodules of the tumor were counted and weighed. All animal use protocols were approved by the Institutional Animal Care and Use Committee of Taipei Medical University (LAC-2019-0340).

### 4.8. RNA Extraction, cDNA Synthesis, and Quantitative Polymerase Chain Reaction (qPCR) Analysis

Total RNA was isolated from fresh-frozen colorectal cancer cell lines using RNAzol^®^RT following the manufacturer’s protocol (Molecular Research Center, Inc., Cincinnati, OH, USA). Subsequently, 8 μg of total RNA was subjected to reverse transcription (RT) reactions in a 20 μL reaction volume using a cDNA Synthesis Kit (Invitrogen Life Technologies, Carlsbad, CA, USA). The resulting cDNA was utilized for quantitative RT-PCR analysis of gene expression employing the Power SYBR-Green real-time RT-PCR system and the ABI 7500 FAST™ detection system (Applied Biosystems, Foster City, CA, USA). The quantification of target gene expression was normalized to the expression levels of the *GAPDH* gene. The primer sequences used for the analysis are presented in Table 1.

### 4.9. Protein Extraction and Western Blot Assay

For protein extraction from CRC cell lines, a total of 1 × 10^6^ cells were seeded into 10 cm culture dishes. The resulting cell lysates were separated using sodium dodecyl sulfate polyacrylamide gel electrophoresis (SDS-PAGE) and subsequently transferred onto polyvinylidene fluoride membranes (GE Healthcare, Piscataway, NJ, USA) for subsequent antibody blotting. The membranes were then incubated with primary antibodies targeting CWH43 (Thermo Fisher Scientific, Waltham, MA, USA BS-9959R), GAPDH (IR3-8, iReal Biotechnology, Inc.), β-catenin (sc-7963, Santa Cruz Biotechnology, Santa Cruz, CA, USA), N-cadherin (13116, Cell Signaling Technology, Danvers, MA, USA), vimentin (iR45-137, iReal Biotechnology Co., Hsinchu City, Taiwan), E-cadherin (3195, Cell Signaling Technology, Danvers, MA, USA) at 4 °C overnight. Following this, the membranes were probed with corresponding secondary antibodies. The protein bands were visualized using an enhanced chemiluminescence reagent (GE Healthcare, Piscataway, NJ, USA) and were captured using the VersaDoc 5000 system (Bio-Rad Laboratories, Hercules, CA, USA).

### 4.10. RNA Sequencing

RNA sequencing was performed as described before [19]. Total RNA was extracted from colorectal cancer (CRC) cells using Trizol (Invitrogen, Carlsbad, CA, USA) following the manufacturer’s protocol. Biotools biotech Co., Ltd. (New Taipei City, Taiwan) performed the RNA sequencing. In brief, ribosomal RNA was depleted from the RNA samples using the EpicentreRibo-Zero rRNA Removal Kit (Illumina, San Diego, CA, USA), after which cDNA synthesis, adaptor ligation, and enrichment steps were executed in accordance with the instructions provided by the NEBNext^®^ Ultra™ RNA Library Prep Kit for Illumina (NEB, Ipswich, MA, USA). The resultant library products were assessed using Illumina NovaSeq 6000 (Illumina, San Diego, CA, USA). with paired-end 150 bp sequencing. Raw reads obtained from sequencing were subjected to quality filtering using Trimmomatic to obtain a set of clean reads. Subsequently, the clean reads were aligned to the reference genome using HISAT2, and the raw read counts for each gene were determined using the feature Counts. For the normalization of expression levels, RLE/TMM/FPKM methods were employed. Differentially expressed genes were identified utilizing a two-fold change threshold with an adjusted *p*-value below 0.05. Expression data from RNA-seq analysis can be found in Table S5 for the CWH43-knockdown condition, and in Table S6 for the CWH43-overexpression condition.

### 4.11. PPI Network Analysis

PPI networks were established using the STRING database (http://www.string-db.org/, accessed on 29 December 2022) [50]. This resource offers both confirmed and predicted protein interactions derived from multiple sources, including genomic contexts, co-expressions, high-throughput experiments, and prior knowledge. A significance threshold of 0.4 (medium confidence) was chosen for screening. The resulting PPI pairs were imported into the Cytoscape software (version 3.8.2), and subsequent analysis was conducted using the CytoNCA plugin (http://www.cytoscape.org, accessed on 29 December 2022) [51]. Hub genes, which represent highly interconnected genes, were identified by calculating their degree value (number of edges connecting the genes), employing a cutoff of ≥5 for the CWH43 knockdown group and ≥88 for the CWH43 overexpression group.

### 4.12. Statistical Analysis

Each experiment was performed independently in triplicate. All data were expressed as the mean ± standard deviation (SD). The data depicted in select figures represent a representative experiment with quantitative consistency to replicate experiments. For comparisons between two groups of datasets, statistical significance was assessed using a two-tailed Student’s *t*-test. Asterisks in the figures denote significant distinctions between the specified experimental groups and their corresponding control conditions.

## Figures and Tables

**Table 1:** Primer sequences for qPCR.

Gene Name	Primer Sequences (5′ → 3′)
*CWH43*	Forward: 5′-CCTCCTTCCAGGCTCCAAAT-3′
	Reverse: 5′-GACACCCCAAGCGCAAGA-3′
*TTK*	Forward: 5′-GCTTGTCAGTTGTCAACACCTTATG-3′
	Reverse: 5′-GGCAAGTATTTGATGCTGTTGCT-3′

## Data Availability

The dataset supporting the conclusions of this article is included within the article.

## Supplementary Materials

The following supporting information can be downloaded at: https://www.mdpi.com/article/10.3390/ijms242015262/s1.

## Abbreviations

Cell Wall Biogenesis 43 C-Terminal Homolog (*CWH43*); colorectal cancer (CRC); epithelial–mesenchymal transition (EMT), microsatellite instability (MSI), Glycosylphosphatidylinositol (GPI).
