# Supplementary material for: CWH43 Is a Novel Tumor Suppressor Gene with Negative Regulation of TTK in Colorectal Cancer
Source: Int J Mol Sci. 2023 Oct 17;24(20):15262. doi: 10.3390/ijms242015262 (PMC10607595; doi:10.3390/ijms242015262)
Supplement: Supplementary file 1 [file ijms-24-15262-s001.zip › ijms-2620166-supplementary.pdf]

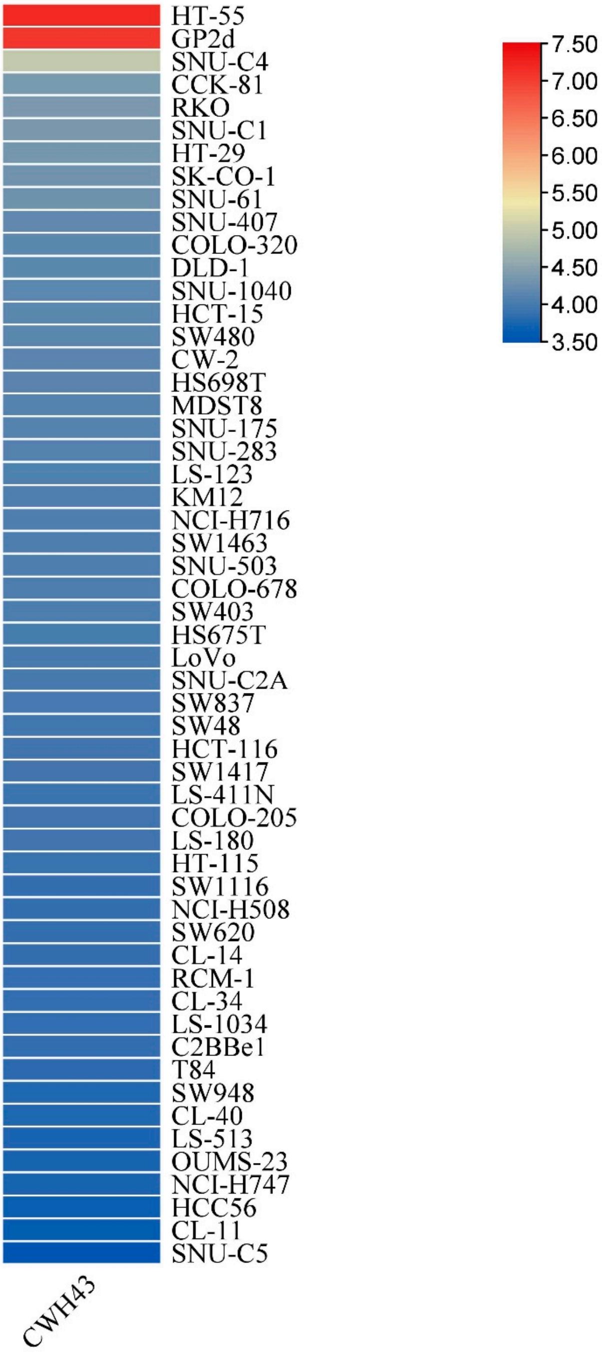

Figure S1 An analysis of CWH43 gene expression in colorectal cancer cell lines based on the CellExpress database (GSE36133), where color gradients from red to blue indicate a descending expression pattern.

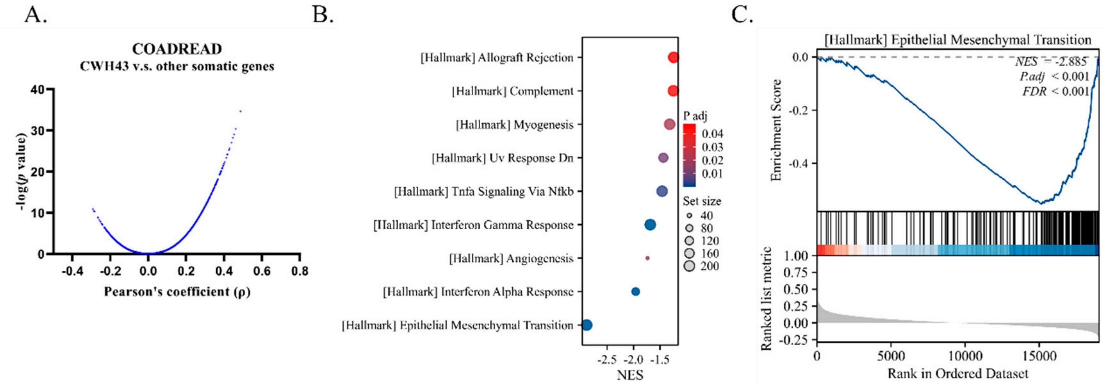

Figure S2 GSEA enrichment analysis of CWH43 co-expressed genes in CRC patients. (A) Volcano plot of CWH43 co-expressed genes. (B) Bubble plot of GSEA results for negatively correlated genes using the Hallmark collections of the GSEA MSigDB. (C) GSEA enrichment plot for epithelial mesenchymal transition.

Table S1. List of differentially expressed genes (DEGs) between the CWH43 KD and scrambled control cells. (A) 107 Up-regulated DEGs. (B) 144 Down-regulated DEGs.

A.

Rank	Gene Symbol	*P* Value	logFC
1	MEF2C	2.49E-02	18.68
2	TC2N	3.93E-02	18.06
3	MSH2	2.63E-02	17.13
4	RPAP3	2.39E-02	17.00
5	SPPL2A	2.11E-02	16.84
6	ZFAND1	3.18E-02	16.73
7	TCEA1	1.89E-02	16.69
8	MED21	2.57E-02	16.63
9	ORC3	3.52E-02	16.62
10	OCRL	3.13E-02	16.58
11	PSEN1	2.61E-02	16.49
12	EHBP1	1.83E-02	16.41
13	RGPD8	3.96E-02	16.32
14	RBL1	2.57E-02	16.25
15	TMEM41B	1.30E-02	16.25
16	TBP	3.58E-02	16.23
17	PHTF2	1.82E-02	16.22
18	CBWD2	4.30E-02	16.22
19	SNRPB2	2.43E-02	16.21
20	ZNF184	4.87E-02	16.01
21	TMEM106C	2.72E-02	15.93
22	LGR4	1.63E-02	15.90
23	PPP4R3A	3.50E-02	15.81
24	RBBP8	2.65E-02	15.79
25	FAM175A	4.86E-02	15.75
26	SERAC1	3.83E-02	15.73
27	CEP135	3.99E-02	15.63
28	CENPE	4.31E-02	15.63
29	ATP2B1	4.44E-02	15.61
30	AGFG1	3.33E-02	15.58
31	SMARCA1	2.72E-02	15.53
32	ACSL4	4.59E-02	15.51
33	PTER	2.09E-02	15.34

34	CXorf57	5.00E-02	15.29
35	CDKAL1	2.95E-02	15.19
36	SLC9A6	4.75E-02	15.15
37	PARK7	3.22E-02	15.13
38	NOMO3	3.04E-02	14.82
39	E2F3	4.23E-02	14.79
40	ARHGAP11A	4.73E-02	14.78
41	HIBADH	3.02E-02	14.71
42	SUSD3	3.41E-02	14.69
43	PLCB1	4.03E-02	14.63
44	ZFHX4	3.56E-02	14.60
45	ATP7A	4.29E-02	14.40
46	IBTK	4.62E-02	14.37
47	FERMT1	3.43E-02	14.36
48	CDKN3	4.21E-02	14.35
49	CEP290	3.78E-02	14.32
50	LANCL1	3.48E-02	14.30
51	CLSPN	3.93E-02	14.27
52	ZBTB43	3.63E-02	14.26
53	BSDC1	4.91E-02	14.26
54	ALOXE3	4.12E-02	14.23
55	VWA9	2.78E-02	14.19
56	TRIM68	4.07E-02	14.18
57	SLC7A8	4.04E-02	14.12
58	C1orf198	2.67E-02	14.04
59	MORC3	4.48E-02	14.02
60	DTNBP1	7.45E-03	13.92
61	POMGNT2	3.25E-02	13.81
62	CCDC138	4.08E-02	13.71
63	SLC9B2	4.14E-02	13.71
64	ZNF761	4.47E-02	13.71
65	SH3KBP1	4.00E-02	13.71
66	GTF3C5	2.88E-02	13.62
67	EIF2B3	3.50E-02	13.51
68	DNAAF2	3.96E-02	13.49
69	PAPOLG	4.96E-02	13.48
70	TBC1D2	2.65E-02	13.47
71	PIGU	3.53E-02	13.37

72	EXOC8	4.95E-02	13.22
73	SMYD2	3.17E-02	13.20
74	TFCP2	4.89E-02	13.10
75	CFL2	3.98E-02	13.09
76	RASSF2	4.35E-02	12.79
77	PDE2A	5.00E-02	12.68
78	FAM214B	4.23E-02	12.49
79	FBXO25	3.30E-02	12.32
80	DNAJB12	4.29E-02	12.18
81	ING4	4.97E-02	12.02
82	CCDC68	4.07E-02	11.93
83	DLGAP5	4.93E-02	4.17
84	ARRDC4	2.51E-02	3.89
85	ARRDC3	1.79E-02	3.69
86	TTK	1.90E-02	3.62
87	CKAP2	3.53E-02	3.45
88	KIF14	2.50E-02	3.34
89	SAMD9	2.69E-02	3.32
90	BBS10	2.59E-02	3.28
91	BRCA2	3.07E-02	3.27
92	ETAA1	2.92E-02	3.20
93	PAIP2B	3.24E-02	3.08
94	CCDC186	3.83E-02	3.03
95	ENPP4	3.31E-02	2.99
96	CXADR	4.77E-02	2.94
97	NEK2	4.23E-02	2.93
98	FNBP1L	1.22E-02	2.90
99	PCGF5	4.35E-02	2.85
100	PRKAA2	4.35E-02	2.84
101	KLF12	3.66E-02	2.81
102	MAL2	1.98E-02	2.79
103	PJA2	4.26E-02	2.73
104	CENPF	2.32E-02	2.70
105	TOP2A	3.16E-02	2.51
106	RLF	4.23E-02	2.42
107	ARL5B	4.51E-02	2.32

B.

Rank	Gene Symbol	*P* Value	logFC
1	ADAM11	1.75E-02	-18.05
2	YDJC	2.36E-02	-17.20
3	SDC1	2.52E-02	-17.10
4	CDIPT	1.70E-02	-17.01
5	WWC3	1.95E-02	-16.69
6	PANK4	2.66E-02	-16.52
7	SAC3D1	1.87E-02	-16.36
8	HS3ST3B1	4.38E-02	-16.30
9	NAT8L	4.22E-02	-16.04
10	KCNH2	2.87E-02	-15.87
11	NTMT1	3.73E-02	-15.85
12	ULK3	3.20E-02	-15.68
13	ESRRA	3.37E-02	-15.63
14	ZNF554	4.67E-02	-15.62
15	CALD1	2.11E-02	-15.62
16	PRDM1	4.17E-02	-15.60
17	TOM1	3.93E-02	-15.57
18	SNX11	2.78E-02	-15.55
19	DHRS7B	2.07E-02	-15.46
20	STON2	1.88E-02	-15.40
21	C16orf59	1.96E-02	-15.40
22	GRTP1	3.05E-02	-15.39
23	WDR5	3.54E-02	-15.35
24	IGHMBP2	2.81E-02	-15.29
25	FAM21C	4.59E-02	-15.03
26	NLRP2	3.38E-02	-15.03
27	WIPI2	3.21E-02	-14.90
28	PROM2	2.68E-02	-14.79
29	LGI3	3.06E-02	-14.77
30	ABL2	4.81E-02	-14.76
31	SUMO3	3.44E-02	-14.74
32	COL11A2	4.79E-02	-14.67
33	LMF2	3.53E-02	-14.63
34	PSMD9	3.33E-02	-14.59
35	TBC1D22A	4.65E-02	-14.50
36	PLEKHM3	4.21E-02	-14.48

37	WDR91	4.58E-02	-14.45
38	WARS2	2.98E-02	-14.43
39	PANX1	2.82E-02	-14.36
40	METTL16	4.50E-02	-14.32
41	NEK3	3.70E-02	-14.27
42	PCYOX1L	3.07E-02	-14.24
43	TOMM40L	3.91E-02	-14.15
44	TP53BP2	4.14E-02	-14.00
45	C12orf49	3.61E-02	-13.95
46	FRMD5	4.21E-02	-13.94
47	CLCN5	4.01E-02	-13.84
48	RPP40	2.86E-02	-13.82
49	TRIM74	4.02E-02	-13.78
50	ZNF655	3.57E-02	-13.71
51	MFAP3L	3.65E-02	-13.63
52	KIF16B	4.93E-02	-13.63
53	AK8	3.81E-02	-13.55
54	PRPSAP2	4.63E-02	-13.48
55	USP48	2.89E-02	-13.39
56	RASGRP2	2.89E-02	-13.23
57	SFR1	3.47E-02	-13.21
58	CCNYL1	4.98E-02	-12.93
59	SDHAF3	2.65E-02	-12.91
60	LHX6	4.33E-02	-12.41
61	CYP4F12	4.13E-02	-12.10
62	PGBD1	3.83E-02	-12.09
63	CYP24A1	3.75E-03	-5.06
64	PRKCDBP	4.71E-02	-4.16
65	MSX1	3.75E-03	-3.61
66	RRS1	2.37E-02	-3.59
67	ANKRD33B	1.48E-02	-3.57
68	CEBPB	3.40E-03	-3.53
69	ZNHIT2	1.74E-02	-3.49
70	NPW	4.40E-03	-3.47
71	IGFBP6	1.27E-02	-3.45
72	COL17A1	2.13E-02	-3.45
73	PHLDA2	4.05E-03	-3.39
74	ZNF579	1.81E-02	-3.38

75	SUSD2	4.09E-02	-3.38
76	ZNF469	6.65E-03	-3.37
77	WNT9A	5.60E-03	-3.33
78	ABCA7	4.38E-02	-3.33
79	TNFRSF18	1.69E-02	-3.33
80	TFAP2E	2.32E-02	-3.31
81	CAPN15	4.53E-02	-3.29
82	ARHGAP39	3.66E-02	-3.16
83	TICAM1	7.20E-03	-3.14
84	MRPL41	7.60E-03	-3.13
85	IRX4	2.93E-02	-3.11
86	TMEM151A	3.18E-02	-3.09
87	MAP1S	2.06E-02	-3.08
88	RHOD	1.05E-02	-3.06
89	CPLX1	3.58E-02	-3.03
90	SCRIB	3.64E-02	-3.01
91	SCAF1	1.00E-02	-3.00
92	SURF6	1.36E-02	-2.97
93	NDUFB7	1.48E-02	-2.95
94	NRTN	3.53E-02	-2.92
95	ZNF696	4.85E-02	-2.92
96	CSNK1G2	4.30E-02	-2.91
97	SELENOM	4.15E-02	-2.91
98	ZNF787	4.44E-02	-2.91
99	MYPOP	4.04E-02	-2.91
100	ZNF628	4.69E-02	-2.90
101	INPP5E	1.42E-02	-2.86
102	TMEM160	3.96E-02	-2.86
103	SP6	4.32E-02	-2.84
104	WNT3A	3.95E-02	-2.83
105	REXO1	4.41E-02	-2.82
106	ZNF865	1.55E-02	-2.80
107	CPTP	3.25E-02	-2.79
108	CDR2L	1.84E-02	-2.77
109	CILP2	2.09E-02	-2.77
110	TUBB2A	2.23E-02	-2.74
111	ZFPM1	1.93E-02	-2.74
112	TOR4A	1.95E-02	-2.74

113	ARC	1.87E-02	-2.73
114	COL18A1	4.97E-02	-2.72
115	SBNO2	2.32E-02	-2.67
116	HIST1H2AI	2.45E-02	-2.65
117	CDKN1A	4.60E-02	-2.63
118	CYBA	4.77E-02	-2.62
119	ZC3H3	2.69E-02	-2.60
120	CCDC86	3.08E-02	-2.58
121	IRF2BPL	2.51E-02	-2.57
122	TRMT61A	3.45E-02	-2.54
123	CPNE7	2.87E-02	-2.53
124	SNAPC4	3.29E-02	-2.50
125	TNS4	3.11E-02	-2.48
126	CENPB	3.49E-02	-2.47
127	PIM3	3.56E-02	-2.46
128	PRR12	4.26E-02	-2.42
129	B4GALNT4	3.47E-02	-2.42
130	TRIM72	4.18E-02	-2.39
131	IER5L	4.36E-02	-2.38
132	C9orf16	3.93E-02	-2.37
133	MMP15	4.56E-02	-2.36
134	JUN	3.96E-02	-2.36
135	RTN4RL2	4.70E-02	-2.33
136	SLC25A22	4.40E-02	-2.33
137	MISP	4.57E-02	-2.31
138	MIER2	4.60E-02	-2.31
139	MLLT1	4.92E-02	-2.30
140	RAB3IL1	4.59E-02	-2.30
141	RBM38	4.50E-02	-2.29
142	C6orf132	4.80E-02	-2.28
143	ATN1	4.72E-02	-2.24
144	PKP3	4.99E-02	-2.20

Table S2. List of differentially expressed genes (DEGs) between the CWH43ov and the control cells. (A) 203 Up-regulated DEGs. (B) 212 Down-regulated DEGs.

A.

Rank	Gene Symbol	*P* Value	logFC
1	SLC5A1	8.40E-08	5.44
2	NR4A3	1.22E-09	5.22
3	CXCL8	3.40E-09	4.80
4	LURAP1L	1.18E-06	4.43
5	TACSTD2	2.12E-09	4.30
6	APOBEC3G	1.48E-06	4.10
7	GTPBP2	3.07E-113	3.96
8	SNTB1	2.78E-15	3.69
9	TRIB3	2.03E-29	3.26
10	NT5E	2.43E-45	3.24
11	KRT80	4.25E-05	3.16
12	DUSP5	5.40E-122	3.14
13	CHAC1	4.05E-08	3.14
14	BHLHE40	2.37E-45	3.13
15	HERPUD1	2.91E-57	3.06
16	DDIT3	5.88E-06	3.02
17	ATF3	1.59E-27	3.00
18	GDF15	2.44E-81	2.96
19	SLC7A11	2.94E-64	2.94
20	NR4A2	5.30E-36	2.94
21	PLAU	5.63E-26	2.92
22	KLF4	1.13E-23	2.79
23	EGR3	4.25E-05	2.69
24	CSRNP1	3.59E-11	2.68
25	GADD45B	5.19E-08	2.64
26	ARG2	4.14E-03	2.60
27	DDIT4	1.36E-09	2.56
28	HBEGF	8.53E-06	2.55
29	HSPA5	0.00E+00	2.54
30	UPP1	7.67E-05	2.46
31	ARL14EPL	2.71E-04	2.46
32	MXD1	5.65E-04	2.39
33	MAFF	7.77E-08	2.38
34	PCK2	2.94E-15	2.38

35	SESN2	6.68E-10	2.31
36	ATF5	2.73E-04	2.30
37	DNAJB9	1.79E-05	2.28
38	STC2	4.95E-20	2.26
39	HTR7	1.95E-08	2.24
40	PKIB	1.08E-07	2.24
41	BIRC3	4.48E-03	2.20
42	PSAT1	2.74E-61	2.18
43	CLIP4	1.21E-08	2.16
44	CYR61	1.36E-26	2.11
45	PPP1R15A	5.33E-20	2.09
46	JDP2	1.08E-04	2.08
47	SLFN5	8.10E-20	2.06
48	KLF6	5.00E-51	2.05
49	SDC4	5.62E-35	2.05
50	HES1	1.05E-14	2.04
51	SMAD3	2.11E-37	2.03
52	FOSL1	2.27E-18	2.03
53	PLAUR	1.10E-07	2.01
54	WNT16	7.05E-21	2.00
55	WARS	1.89E-27	1.99
56	DHRS3	8.51E-08	1.99
57	CD55	1.36E-09	1.98
58	IRS1	2.05E-32	1.96
59	NR4A1	5.33E-21	1.94
60	TSC22D3	1.74E-03	1.92
61	FAM84B	1.88E-18	1.92
62	THBS1	6.96E-06	1.90
63	CDKN1A	8.09E-30	1.90
64	STARD4	2.88E-09	1.85
65	AREG	8.70E-74	1.83
66	FAM129A	5.71E-05	1.81
67	CASP4	9.33E-04	1.81
68	DNAJB11	1.14E-12	1.81
69	LAMB3	5.80E-10	1.79
70	SEMA3A	2.68E-07	1.77
71	FAM3C	1.28E-55	1.77
72	GPRC5A	3.62E-58	1.75

73	VEGFA	3.58E-12	1.75
74	ADGRF1	1.71E-34	1.74
75	APOBEC3F	1.14E-06	1.73
76	SLC7A5	9.68E-80	1.72
77	OCLN	7.74E-06	1.72
78	SLC3A2	1.65E-33	1.70
79	CLDN4	4.42E-19	1.68
80	RPE65	5.75E-05	1.67
81	CASC19	2.69E-06	1.66
82	CEBPG	4.07E-13	1.66
83	HS3ST1	7.49E-12	1.65
84	TNFRSF10B	2.79E-28	1.63
85	UBE2J1	2.50E-09	1.63
86	LDLR	4.62E-17	1.63
87	ARL5B	7.63E-13	1.62
88	NFIL3	4.81E-06	1.62
89	MTND2P28	5.97E-04	1.62
90	DUSP1	2.53E-04	1.61
91	HK2	2.27E-12	1.60
92	KRT23	4.45E-07	1.60
93	CYP24A1	1.69E-20	1.59
94	EMP1	2.12E-08	1.57
95	SIPA1L2	3.21E-04	1.57
96	DNAJC3	2.83E-14	1.57
97	HMGA2	2.05E-34	1.56
98	ACSL5	1.54E-44	1.53
99	TRIM8	2.00E-07	1.51
100	SEL1L	1.69E-15	1.50
101	MANF	3.26E-09	1.49
102	BTG1	3.76E-06	1.48
103	FOSB	1.35E-16	1.48
104	DUSP4	3.68E-24	1.48
105	OR51B4	2.85E-03	1.48
106	S100A14	4.78E-13	1.48
107	PHGDH	2.79E-16	1.48
108	IRF2BP2	4.39E-06	1.47
109	DSC2	5.38E-15	1.47
110	FRMD5	4.34E-06	1.47

111	PARD6B	1.97E-03	1.46
112	TRNL1	3.56E-03	1.46
113	TMEM41B	7.44E-09	1.46
114	CARS	1.08E-12	1.45
115	RBCK1	9.96E-06	1.45
116	OGFRL1	5.03E-05	1.44
117	RUNX1	1.28E-05	1.43
118	EPHA2	2.51E-15	1.42
119	JUN	1.49E-08	1.42
120	HYOU1	1.29E-39	1.41
121	NFKBIA	1.46E-03	1.41
122	ND2	0.00E+00	1.41
123	PLK2	1.95E-11	1.40
124	EXOSC6	7.87E-05	1.38
125	EGR1	1.22E-35	1.37
126	LINC00963	1.51E-04	1.37
127	FAM107B	2.03E-08	1.36
128	SLC38A2	4.11E-134	1.35
129	ERRFI1	1.03E-61	1.35
130	FGF19	3.40E-04	1.34
131	TXNRD1	2.67E-51	1.34
132	MTHFD2	6.14E-30	1.32
133	ARHGEF2	1.36E-05	1.32
134	CBX4	1.33E-05	1.32
135	CHST6	2.60E-03	1.30
136	GPAT3	1.86E-03	1.29
137	BCL10	1.93E-03	1.29
138	SARS	6.03E-14	1.29
139	KDM6B	2.30E-05	1.28
140	SLC7A1	1.29E-16	1.28
141	TMED7	5.16E-10	1.28
142	MTHFD1L	2.38E-09	1.27
143	SH2B3	1.98E-04	1.27
144	AJUBA	3.09E-03	1.26
145	GPT2	2.97E-05	1.26
146	YARS	6.88E-19	1.26
147	PDIA4	2.79E-40	1.26
148	IFRD1	2.43E-03	1.26

149	PMAIP1	1.12E-04	1.25
150	FNDC3B	1.43E-05	1.23
151	GADD45A	5.44E-04	1.23
152	PCDH7	1.19E-13	1.23
153	ND3	1.06E-99	1.22
154	XBP1	1.85E-05	1.22
155	CLDN7	1.82E-04	1.22
156	ZFP36	5.96E-05	1.21
157	SH3KBP1	5.54E-08	1.21
158	ACSS2	3.68E-03	1.21
159	GOT1	4.70E-09	1.20
160	HSP90B1	7.16E-82	1.20
161	ASS1	8.27E-08	1.19
162	TINAGL1	3.28E-03	1.17
163	FXYD5	1.97E-03	1.17
164	CLIC4	1.97E-14	1.17
165	PSPH	4.14E-03	1.17
166	PNRC1	2.86E-03	1.16
167	STK39	6.04E-12	1.16
168	HSPA13	3.51E-04	1.16
169	MEF2D	6.40E-04	1.15
170	TMSB4X	2.89E-08	1.15
171	PHLDA2	1.56E-03	1.14
172	LAMC2	2.84E-03	1.14
173	SERINC1	4.84E-06	1.13
174	RCN1	2.14E-05	1.13
175	ANXA3	4.96E-05	1.12
176	TNFRSF10D	8.21E-05	1.11
177	ITCH	4.05E-08	1.11
178	SRF	1.60E-03	1.11
179	GARS	4.29E-17	1.10
180	CYTB	0.00E+00	1.10
181	CCNB1IP1	4.52E-05	1.10
182	SLC38A1	7.94E-23	1.10
183	PVR	5.59E-04	1.09
184	CDK6	2.58E-10	1.09
185	IER3	3.97E-20	1.08
186	MAP1B	8.24E-05	1.08

187	LIF	1.21E-03	1.08
188	GFPT1	1.82E-08	1.08
189	MALT1	4.41E-05	1.07
190	PRKACB	1.47E-13	1.07
191	ATF6	1.64E-04	1.05
192	PHLDA1	7.10E-27	1.05
193	ATP9A	9.46E-05	1.05
194	AFAP1	2.76E-03	1.04
195	CALR	4.13E-43	1.04
196	ITPRIPL2	5.45E-05	1.04
197	TRIM38	2.73E-03	1.04
198	ZMAT3	1.15E-03	1.03
199	EREG	2.51E-34	1.02
200	PYGB	1.67E-06	1.01
201	VASP	9.58E-05	1.01
202	YIPF6	1.53E-03	1.01
203	ATG12	1.81E-06	1.00

B.

**Rank**	**Gene Symbol**	***P* Value**	**logFC**
1	RPS4Y1	6.49E-05	-8.74
2	VASH2	2.77E-04	-7.32
3	TIMP3	3.30E-04	-5.07
4	DDX3Y	2.97E-03	-4.68
5	PEG10	2.81E-19	-3.89
6	HOXB8	5.81E-07	-3.73
7	AXIN2	6.93E-09	-3.58
8	CEMIP	2.09E-11	-3.39
9	CENPE	2.10E-06	-3.20
10	RGS2	1.60E-03	-2.68
11	MMP14	3.39E-04	-2.63
12	MAP1A	5.31E-04	-2.62
13	NRGN	1.55E-03	-2.61
14	CLU	2.91E-18	-2.56
15	SESN3	5.03E-06	-2.54
16	DPP4	3.13E-03	-2.53
17	GATA2	1.67E-05	-2.36

18	DDIT4L	7.12E-04	-2.34
19	SGO2	1.00E-04	-2.26
20	BRCA2	2.56E-03	-2.24
21	CENPF	6.60E-30	-2.20
22	SAMD5	1.14E-04	-2.20
23	HOXB6	1.10E-03	-2.18
24	BMP4	9.97E-34	-2.16
25	NFIB	4.22E-03	-2.03
26	ASPM	2.26E-08	-2.03
27	ZGRF1	1.02E-03	-1.98
28	DKK1	1.17E-10	-1.92
29	STARD9	3.98E-05	-1.90
30	SPTB	1.10E-03	-1.85
31	TK1	1.13E-17	-1.84
32	KIF20B	1.71E-04	-1.82
33	ERCC6L	1.23E-03	-1.80
34	TBX3	1.17E-03	-1.78
35	MRC2	2.67E-04	-1.78
36	H2AFX	4.29E-09	-1.77
37	LIG1	4.89E-07	-1.75
38	ZWINT	2.78E-06	-1.73
39	NGFR	1.54E-03	-1.70
40	FSD1	3.73E-03	-1.70
41	YBX1	5.18E-109	-1.69
42	NBEAL1	8.82E-04	-1.68
43	NT5DC2	4.91E-08	-1.67
44	GINS2	9.29E-05	-1.67
45	DLGAP5	1.35E-08	-1.65
46	FAM83D	2.97E-05	-1.64
47	CDC20	8.32E-16	-1.64
48	KIF20A	7.31E-07	-1.63
49	GEN1	1.30E-03	-1.63
50	DUT	1.00E-04	-1.62
51	MKI67	2.50E-82	-1.61
52	MCM4	1.62E-29	-1.59
53	KIF5C	2.91E-03	-1.57
54	DENND5B	4.22E-11	-1.56
55	KIF14	3.20E-04	-1.56

56	PRKDC	1.33E-123	-1.56
57	TONSL	1.02E-05	-1.56
58	CEP152	3.80E-03	-1.54
59	NES	2.77E-05	-1.54
60	MYBL2	1.98E-09	-1.54
61	TOP2A	1.30E-25	-1.54
62	SYNE2	8.61E-18	-1.52
63	PBX1	2.10E-05	-1.51
64	FANCG	1.46E-03	-1.50
65	SPCS1	3.44E-04	-1.50
66	MT2A	5.04E-07	-1.50
67	SESTD1	7.18E-06	-1.49
68	RECQL4	4.32E-07	-1.48
69	TIMELESS	7.34E-11	-1.48
70	MARCKS	1.32E-06	-1.47
71	CCDC14	1.34E-09	-1.46
72	MIS18BP1	2.49E-03	-1.46
73	CCNF	4.30E-05	-1.46
74	SMC4	1.40E-10	-1.46
75	BUB1B	2.36E-08	-1.46
76	CCNB1	1.38E-14	-1.45
77	AP1M1	2.70E-03	-1.45
78	SMC2	1.72E-03	-1.45
79	NKD1	2.62E-03	-1.45
80	SLC2A4RG	4.91E-04	-1.45
81	AKR7A2	1.91E-03	-1.44
82	TTK	2.54E-04	-1.44
83	VPS13A	5.16E-14	-1.44
84	CCNA2	7.06E-06	-1.44
85	ETV1	3.16E-04	-1.43
86	COL12A1	1.59E-10	-1.41
87	KIFC1	4.63E-06	-1.41
88	GABBR1	5.47E-05	-1.41
89	MCM2	4.01E-10	-1.39
90	INCENP	2.00E-06	-1.39
91	BIRC5	9.29E-13	-1.39
92	GINS1	2.20E-05	-1.38
93	CDCA2	1.38E-04	-1.38

94	ARID5B	4.94E-05	-1.37
95	HJURP	1.81E-04	-1.37
96	ESCO2	2.86E-03	-1.37
97	TRNP1	6.23E-04	-1.37
98	EPPK1	2.70E-05	-1.37
99	KIF11	3.76E-06	-1.36
100	CBX2	2.53E-04	-1.36
101	ID3	1.57E-08	-1.36
102	POLQ	2.62E-03	-1.36
103	ARF5	3.13E-06	-1.34
104	KNTC1	5.49E-08	-1.33
105	CLSPN	1.55E-05	-1.33
106	CPOX	1.80E-41	-1.32
107	BUB1	6.90E-09	-1.32
108	PASK	2.10E-03	-1.32
109	ESPL1	3.67E-05	-1.32
110	CENPJ	2.59E-03	-1.32
111	MCM5	1.64E-08	-1.31
112	TNS4	2.29E-06	-1.31
113	TACC3	3.69E-09	-1.31
114	CUTC	2.06E-03	-1.30
115	THRA	1.85E-03	-1.30
116	TICRR	1.57E-03	-1.29
117	PLK1	1.26E-06	-1.29
118	PDLIM5	2.67E-35	-1.29
119	BAHCC1	6.27E-06	-1.28
120	RACGAP1	1.21E-09	-1.27
121	TUBA1B	3.31E-19	-1.27
122	CPT1A	1.05E-13	-1.26
123	GTSE1	2.04E-03	-1.26
124	CHTF18	1.03E-03	-1.26
125	CDCA8	2.42E-03	-1.25
126	RFC4	1.70E-03	-1.25
127	NCAPG	1.41E-07	-1.24
128	LMNB1	1.82E-10	-1.24
129	PALLD	1.74E-15	-1.24
130	FANCD2	3.70E-05	-1.24
131	IL17RD	7.57E-05	-1.24

132	KNL1	7.87E-05	-1.24
133	AURKA	1.51E-04	-1.24
134	HMMR	2.50E-03	-1.23
135	BRD8	2.37E-04	-1.23
136	KIF23	2.25E-07	-1.23
137	PAM	1.68E-34	-1.23
138	GRIN2B	1.67E-06	-1.23
139	HMGN2	2.14E-14	-1.23
140	CDK1	1.53E-05	-1.22
141	FLOT1	3.63E-04	-1.22
142	NCAPD2	1.59E-23	-1.22
143	NCAPG2	2.47E-07	-1.22
144	BLM	3.46E-03	-1.20
145	SKP2	1.54E-03	-1.20
146	SHCBP1	1.90E-03	-1.20
147	DEPDC1	2.43E-05	-1.20
148	POLD1	1.96E-03	-1.20
149	CDC25B	2.89E-13	-1.19
150	MRPS34	4.30E-04	-1.19
151	KCNQ2	6.47E-04	-1.19
152	DPYSL2	2.01E-06	-1.19
153	VCAN	6.93E-09	-1.18
154	ASF1B	1.23E-03	-1.18
155	POLA1	2.92E-05	-1.18
156	PDK3	9.54E-04	-1.17
157	RRM1	1.63E-10	-1.17
158	ID2	3.67E-06	-1.17
159	MELK	2.65E-05	-1.17
160	COPG2	1.39E-03	-1.16
161	FOXM1	2.56E-05	-1.15
162	FEN1	1.31E-07	-1.15
163	KREMEN1	1.76E-06	-1.15
164	WDR34	4.14E-03	-1.15
165	MCM6	3.41E-11	-1.14
166	IGFBP2	6.32E-05	-1.14
167	CEP55	8.38E-05	-1.14
168	HMGB2	6.97E-12	-1.13
169	NCAPH	1.54E-04	-1.13

170	COL18A1	2.39E-06	-1.13
171	ACOT7	1.39E-05	-1.13
172	CDCA5	7.69E-04	-1.12
173	DHFR	3.18E-08	-1.12
174	PTMAP5	6.97E-05	-1.12
175	TYMS	1.33E-03	-1.12
176	KIF4A	3.73E-04	-1.12
177	ABCC2	6.91E-22	-1.12
178	STMN3	1.49E-04	-1.12
179	KIF22	8.11E-05	-1.11
180	FABP5	1.31E-05	-1.11
181	TSTA3	1.18E-03	-1.11
182	RPS6KA1	6.93E-04	-1.11
183	MCM10	1.45E-03	-1.11
184	ANLN	2.31E-08	-1.10
185	FANCA	1.02E-04	-1.10
186	SAPCD2	3.01E-03	-1.10
187	TPX2	3.90E-18	-1.10
188	MAPK12	1.39E-03	-1.09
189	RFC2	2.33E-03	-1.09
190	DDX11	1.08E-04	-1.08
191	EPN1	4.96E-05	-1.08
192	MDC1	1.20E-07	-1.08
193	LSM4	8.33E-08	-1.08
194	TPGS2	8.65E-05	-1.08
195	CKS2	9.28E-04	-1.07
196	TUBB4B	3.13E-17	-1.06
197	FAM208A	8.33E-05	-1.05
198	HSPH1	5.99E-12	-1.05
199	AGPAT5	2.64E-09	-1.04
200	OAS3	5.85E-04	-1.04
201	HELLS	8.12E-07	-1.04
202	MTHFD1	1.91E-09	-1.04
203	POLE	6.15E-11	-1.03
204	EIF2AK1	8.53E-13	-1.02
205	GLB1L2	4.09E-03	-1.02
206	TMPO	2.70E-16	-1.02
207	LY6E	7.51E-05	-1.02

208	MCM3	1.30E-12	-1.01
209	SPAG5	8.46E-04	-1.01
210	FANCI	1.99E-06	-1.01
211	HSP90AA1	3.28E-191	-1.00
212	CHAF1A	3.52E-04	-1.00

Table S3. The rank of hub genes between CWH43 KD and scrambled control cells. Hub genes are defined as which DEGs had interactions (Degree ≥ 5) in the protein-protein interaction (PPI) network by CytoNCA.

NO.	Hub genes	Degree	Betweenness	Closeness
1	TOP2A	16	1207.32	0.0177
2	JUN	15	3902.37	0.0178
3	CENPF	15	578.31	0.0176
4	CENPE	15	1073.68	0.0177
5	TBP	14	2736.56	0.0177
6	TTK	14	176.50	0.0176
7	CDKN3	13	603.02	0.0177
8	BRCA2	11	641.07	0.0176
9	KIF14	11	1086.82	0.0177
10	CLSPN	11	328.90	0.0176
11	WDR5	11	3082.92	0.0177
12	NEK2	10	77.76	0.0176
13	DLGAP5	10	9.39	0.0176
14	ARHGAP11A	10	9.39	0.0176
15	CKAP2	9	2.16	0.0176
16	RBL1	8	163.17	0.0177
17	E2F3	8	860.38	0.0177
18	CDKN1A	8	770.14	0.0176
19	MSH2	8	528.69	0.0176
20	WNT3A	7	793.24	0.0176
21	RBBP8	7	31.97	0.0176
22	MEF2C	7	905.11	0.0176
23	PLCB1	6	1514.61	0.0173
24	SURF6	6	1033.59	0.0176
25	CEP135	5	982.56	0.0176
26	AK8	5	348.00	0.0176
27	AGFG1	5	1296.79	0.0176

Table S4. The rank of hub genes between the CWH43ov and the control cells. Hub genes are defined as which DEGs had interactions (Degree ≥ 88) in the protein-protein interaction (PPI) network by CytoNCA.

NO.	Hub genes	Degree	Betweenness	Closeness
1	CDK1	118	4163.40	0.0774
2	CCNB1	109	3733.34	0.0771
3	CCNA2	105	2414.23	0.0770
4	TOP2A	104	1794.31	0.0765
5	AURKA	102	2743.54	0.0765
6	BUB1B	102	1307.35	0.0763
7	PLK1	102	1379.27	0.0766
8	BUB1	102	671.41	0.0756
9	KIF11	100	1228.61	0.0757
10	CDC20	100	573.39	0.0758
11	MCM2	97	513.59	0.0757
12	MCM3	97	726.97	0.0758
13	ASPM	97	1964.67	0.0754
14	NCAPG	96	282.29	0.0753
15	KIF20A	96	691.51	0.0756
16	MCM4	94	635.78	0.0759
17	KIF23	93	671.63	0.0754
18	TTK	93	921.16	0.0752
19	MCM5	92	1013.11	0.0755
20	NCAPH	91	235.02	0.0753
21	BIRC5	91	2012.97	0.0762
22	KIF4A	91	129.71	0.0752
23	CENPF	91	1077.24	0.0757
24	TPX2	90	713.81	0.0756
25	FEN1	90	1334.96	0.0757
26	SMC4	89	166.74	0.0752
27	CENPE	88	368.85	0.0750
28	MCM10	88	170.64	0.0753

Table S5. The relevant expression value of differentially expressed genes (DEGs) in the CWH43 KD cells and the scrambled control cells.

Gene symbol	Control	CWH43-KD
ABCA7	63.10180	6.27526
ABL2	12.83630	0.00046
ACN9	3.35514	0.00043
ACSL4	0.00017	7.84135
ADAM11	7.10016	0.00003
AGFG1	0.00042	20.66210
AK8	3.67208	0.00031
ALOXE3	0.00015	2.95426
ANKRD33B	59.29220	5.00946
ARC	31.80530	4.80107
ARHGAP11A	0.00026	7.28876
ARHGAP39	44.41130	4.96483
ARL5B	2.46780	12.32660
ARRDC3	2.24996	29.02170
ARRDC4	0.71937	10.62990
ATN1	128.42600	27.17810
ATP2B1	0.00017	8.44369
ATP7A	0.00020	4.38967
B4GALNT4	52.35300	9.78683
BBS10	1.09448	10.65750
BRCA2	0.41584	4.00304
BSDC1	0.00075	14.58300
C10orf118	1.05478	8.61737
C12orf49	30.21700	0.00191
C16orf59	12.40820	0.00029
C1orf198	0.00038	6.48229
C6orf132	24.45270	5.02843
C9orf16	315.11800	60.92330
CALD1	19.93630	0.00040
CAPN15	48.24520	4.91844
CBWD2	0.00008	6.11184
CCDC138	0.00015	2.03193
CCDC68	0.00042	1.63891
CCDC86	176.17900	29.40430

CCNYL1	3.33060	0.00043
CDIPT	51.22500	0.00039
CDKAL1	0.00014	5.36905
CDKN1A	729.59700	117.66500
CDKN3	0.00070	14.67570
CDR2L	84.69270	12.40430
CEBPB	134.09300	11.61130
CENPB	120.65900	21.75180
CENPE	0.00008	3.95470
CENPF	4.97957	32.40570
CEP135	0.00003	1.76822
CEP290	0.00011	2.24230
CFL2	0.00124	10.74740
CILP2	37.03460	5.42914
CKAP2	2.05649	22.46940
CLCN5	1.90619	0.00013
CLSPN	0.00020	3.98543
COL11A2	2.49308	0.00010
COL17A1	5.51289	0.50538
COL18A1	78.09770	11.87180
CPLX1	19.53320	2.39080
CPNE7	148.27500	25.76140
CSNK1G2	160.07900	21.27290
CXADR	1.36851	10.51980
CXorf57	0.00007	2.61632
CYBA	481.48400	78.28880
CYP24A1	73.13700	2.19544
CYP4F12	2.77184	0.00063
DHRS7B	8.81562	0.00020
DLGAP5	1.17991	21.29540
DNAAF2	0.00017	1.94472
DNAJB12	0.00062	2.87627
DTNBP1	0.00035	5.36352
E2F3	0.00022	6.14800
EHBP1	0.00017	14.38100
EIF2B3	0.00077	8.98535
ENPP4	0.75581	6.00170
ESRRA	27.81470	0.00055

ETAA1	0.68575	6.30566
EXOC8	0.00029	2.78676
FAM175A	0.00014	7.97764
FAM214B	0.00069	3.97843
FAM21C	0.00053	2.74795
FBXO25	0.00067	3.40223
FERMT1	0.00037	7.80509
FNBP1L	2.16526	16.18730
FRMD5	10.90020	0.00069
GLTPD1	47.59130	6.85881
GRTP1	5.76593	0.00013
GTF3C5	0.00138	17.40580
HIBADH	0.00045	12.11920
HIST1H2AI	365.89400	58.36530
HS3ST3B1	3.07519	0.00004
IBTK	0.00043	9.08566
IER5L	143.76000	27.67110
IGFBP6	270.04300	24.68310
IGHMBP2	7.60289	0.00019
ING4	0.00066	2.73657
INPP5E	38.00080	5.23529
IRF2BPL	133.39800	22.45710
IRX4	20.85740	2.42240
JUN	70.64130	13.73560
KCNH2	22.80320	0.00038
KIF14	0.73707	7.46471
KIF16B	1.56006	0.00012
KLF12	0.65881	4.61036
LANCL1	0.00042	8.37592
LGI3	3.69680	0.00013
LGR4	0.00007	4.16527
LHX6	3.06527	0.00056
LMF2	20.13020	0.00079
MAL2	9.60425	66.43330
MAP1S	65.76120	7.77066
MED21	0.00009	9.15247
MEF2C	0.00002	8.57248
METTL16	5.74764	0.00028

MFAP3L	1.36793	0.00011
MIER2	53.21450	10.76800
MISP	104.06600	20.94040
MLLT1	57.88880	11.72580
MMP15	63.80760	12.40090
MORC3	0.00045	7.50720
MRPL41	170.48000	19.48850
MSH2	0.00012	16.64630
MSX1	303.87900	24.94190
MYPOP	33.39460	4.45739
NAT8L	5.41122	0.00008
NDUFB7	469.13300	60.50710
NEK2	2.77485	21.13400
NEK3	2.28552	0.00012
NLRP2	32.33070	0.00097
NOMO3	0.00022	6.26908
NPW	515.84000	46.52430
NRTN	20.80220	2.74602
NTMT1	22.45540	0.00038
OCRL	0.00011	10.54560
ORC3	0.00012	12.37160
PAIP2B	0.66733	5.66223
PANK4	21.06870	0.00022
PANX1	3.86788	0.00018
PAPOLG	0.00031	3.53543
PARK7	0.00168	59.96670
PCGF5	0.88378	6.37812
PCYOX1L	4.79751	0.00025
PDE2A	0.00054	3.52449
PGBD1	1.62253	0.00037
PHLDA2	761.10000	72.36980
PHTF2	0.00009	6.88327
PIGU	0.00110	11.69500
PIM3	187.80600	34.05630
PJA2	2.52303	16.68730
PKP3	164.90200	35.90340
PLCB1	0.00008	1.94148
PLEKHM3	2.72076	0.00012

POMGNT2	0.00034	4.94376
PRDM1	2.20982	0.00004
PRKAA2	0.43050	3.08646
PRKCDBP	35.66130	1.99598
PROM2	8.16114	0.00029
PRPSAP2	8.48499	0.00074
PRR12	45.45950	8.48833
PSEN1	0.00012	10.63410
PSMD9	30.64450	0.00125
PTER	0.00006	2.43953
RAB3IL1	35.17740	7.14988
RASGRP2	8.68854	0.00091
RASSF2	0.00032	2.24354
RBBP8	0.00010	5.92911
RBL1	0.00005	3.81432
RBM38	51.98910	10.61200
REXO1	64.36320	9.11344
RGPD8	0.00002	1.74895
RHOD	186.56000	22.37400
RLF	4.10256	21.91790
RPAP3	0.00007	8.64789
RPP40	3.30772	0.00023
RRS1	89.63970	7.42107
RTN4RL2	55.63930	11.08690
SAC3D1	23.37450	0.00028
SAMD9	0.53983	5.38978
SBNO2	76.35790	12.00850
SCAF1	163.59000	20.41360
SCRIB	222.45700	27.66520
SDC1	26.11660	0.00019
SELM	204.51600	27.22580
SERAC1	0.00004	2.27119
SFR1	3.32965	0.00035
SH3KBP1	0.00080	10.62470
SLC25A22	85.45180	17.04960
SLC7A8	0.00024	4.31102
SLC9A6	0.00011	4.07082
SLC9B2	0.00014	1.82477

SMARCA1	0.00031	14.48940
SMEK1	0.00017	9.80409
SMYD2	0.00063	5.97850
SNAPC4	43.23190	7.63666
SNRPB2	0.00012	9.32975
SNX11	10.36920	0.00022
SP6	20.52480	2.86792
SPPL2A	0.00006	6.81888
STON2	4.69625	0.00011
SUMO3	10.74360	0.00039
SURF6	55.76280	7.11987
SUSD2	15.79160	1.52004
SUSD3	0.00028	7.37342
TBC1D2	0.00057	6.43008
TBC1D22A	16.44470	0.00071
TBP	0.00007	5.62025
TC2N	0.00003	7.76613
TCEA1	0.00017	17.96660
TFAP2E	12.17870	1.23157
TFCP2	0.00071	6.28157
TICAM1	74.82240	8.48208
TMEM106C	0.00033	20.84110
TMEM151A	24.08320	2.82768
TMEM160	45.84420	6.32317
TMEM41B	0.00011	8.70762
TNFRSF18	91.67800	9.13057
TNS4	76.61080	13.74670
TOM1	8.62249	0.00018
TOMM40L	8.71915	0.00048
TOP2A	6.02806	34.43960
TOR4A	74.69190	11.20520
TP53BP2	4.74867	0.00029
TRIM68	0.00014	2.67513
TRIM72	57.04380	10.87910
TRIM74	9.36405	0.00066
TRMT61A	21.23570	3.64651
TTK	1.94386	23.87980
TUBB2A	83.55210	12.49240

ULK3	21.74040	0.00041
USP48	3.94789	0.00037
VWA9	0.00034	6.36502
WARS2	3.84217	0.00017
WDR5	23.46640	0.00056
WDR91	2.73105	0.00012
WIPI2	31.15680	0.00102
WNT3A	12.03490	1.69479
WNT9A	32.90330	3.26701
WWC3	8.24235	0.00008
YDJC	32.87550	0.00022
ZBTB43	0.00016	3.06454
ZC3H3	59.70520	9.86019
ZFAND1	0.00013	13.91530
ZFHX4	0.00010	2.43917
ZFPM1	15.41850	2.31060
ZNF184	0.00006	3.69806
ZNF469	35.14740	3.40073
ZNF554	2.23714	0.00004
ZNF579	71.23650	6.83547
ZNF628	19.44070	2.61202
ZNF655	3.36017	0.00025
ZNF696	32.10870	4.25625
ZNF761	0.00026	3.44908
ZNF787	58.76900	7.83845
ZNF865	38.40390	5.49536
ZNHIT2	33.87960	3.01863

Table S6. The relevant expression value of differentially expressed genes (DEGs) in the CWH43 ov cells and the control cells.

Gene symbol	Control	CWH43ov
ABCC2	396.98611	183.08384
ACOT7	98.40148	45.10017
ACSL5	261.90317	757.85405
ACSS2	32.98828	76.15648
ADGRF1	142.65710	477.37669
AFAP1	51.26642	105.50013
AGPAT5	185.78599	90.25742
AJUBA	29.92106	71.87482
AKR7A2	41.43879	15.24271
ANLN	154.17483	71.93191
ANXA3	67.29109	146.48991
AP1M1	38.93494	14.21512
APOBEC3F	27.91798	92.65515
APOBEC3G	1.94049	33.33987
AREG	263.34288	938.54016
ARF5	86.63336	34.13911
ARG2	3.75578	22.77844
ARHGEF2	49.01295	122.11298
ARID5B	67.16589	25.91832
ARL14EPL	6.38483	35.16671
ARL5B	64.78723	199.75377
ASF1B	57.58865	25.34743
ASPM	69.66975	17.06956
ASS1	91.07770	207.63203
ATF3	23.09806	185.02486
ATF5	7.76195	38.30660
ATF6	71.17206	147.57459
ATG12	119.05828	238.23164
ATP9A	75.92938	157.05134
AURKA	69.41936	29.45783
AXIN2	43.12889	3.59660
BAHCC1	87.69750	36.08013
BCL10	30.60962	74.72926
BHLHE40	33.23867	291.15297

BIRC3	6.07185	27.91643
BIRC5	172.20258	65.82341
BLM	48.26179	20.95160
BMP4	260.90162	58.23059
BRCA2	23.16065	4.90964
BRD8	66.53993	28.31605
BTG1	39.24792	109.72471
BUB1	128.07215	51.32285
BUB1B	105.22448	38.36369
CALR	723.30093	1485.79356
CARS	86.82115	237.71784
CASC19	29.48289	93.22604
CASP4	13.39562	46.92701
CBX2	57.77644	22.49299
CBX4	49.20074	122.51260
CCDC14	120.93617	43.95839
CCNA2	74.42707	27.45972
CCNB1	185.16003	67.59316
CCNB1IP1	71.17206	152.82676
CCNF	62.78415	22.83553
CD55	27.98057	110.01015
CDC20	172.20258	55.43324
CDC25B	220.21399	96.42301
CDCA2	60.28029	23.12097
CDCA5	66.03916	30.31416
CDCA8	48.32439	20.26653
CDK1	86.94634	37.22191
CDK6	154.30003	327.51855
CDKN1A	96.58618	361.02968
CEBPG	62.53376	197.35604
CEMIP	57.65125	5.48053
CENPE	33.48905	3.65368
CENPF	227.60036	49.61018
CENPJ	44.69380	17.92589
CEP152	34.11502	11.70321
CEP55	82.87758	37.73571
CHAC1	5.88406	51.89374
CHAF1A	84.69287	42.24572

CHST6	28.35615	69.99089
CHTF18	54.33364	22.72135
CKS2	69.16898	32.99734
CLDN4	88.19827	282.01876
CLDN7	47.76102	110.92357
CLIC4	178.02405	399.96425
CLIP4	19.46747	87.06045
CLSPN	77.61949	30.88505
CLU	118.43231	20.03817
COL12A1	138.33795	51.89374
COL18A1	111.98489	51.20867
COPG2	58.52760	26.26086
CPOX	592.78752	236.69024
CPT1A	209.63521	87.23171
CSRNP1	13.33302	85.46196
CUTC	46.82208	18.95349
CXCL8	1.56491	43.50168
CYP24A1	109.54363	329.91628
CYR61	64.47425	277.62292
CYTB	7911.86681	16997.45323
DDIT3	4.81992	39.22002
DDIT4	13.20783	77.64079
DDIT4L	26.35307	5.19508
DDX11	86.06999	40.59015
DDX3Y	12.83225	0.00000
DENND5B	128.32254	43.50168
DEPDC1	86.25778	37.62153
DHFR	147.28923	67.65025
DHRS3	22.03392	87.68842
DKK1	95.39685	25.29035
DLGAP5	92.07924	29.28656
DNAJB11	47.44804	166.18555
DNAJB9	10.32840	50.29525
DNAJC3	79.62257	235.77682
DPP4	19.71785	3.42533
DPYSL2	105.53746	46.29903
DSC2	99.65340	275.85317
DUSP1	21.72094	66.10885

DUSP4	156.86648	437.69996
DUSP5	87.57231	774.52399
DUT	50.64045	16.49867
EGR1	283.24852	733.64839
EGR3	5.88406	37.96406
EIF2AK1	262.27874	129.02073
EMP1	46.07092	137.07025
EPHA2	111.17113	297.94654
EPN1	92.83040	43.78712
EPPK1	71.48504	27.74517
ERCC6L	33.30126	9.59092
EREG	599.17235	1218.10410
ERRFI1	512.85197	1305.67834
ESCO2	41.75177	16.15614
ESPL1	72.92476	29.22947
ETV1	52.58094	19.46729
EXOSC6	36.80666	95.96630
FABP5	100.96793	46.81283
FAM107B	69.29417	177.26078
FAM129A	17.96516	63.19732
FAM208A	91.89146	44.24383
FAM3C	218.46129	746.66464
FAM83D	55.71076	17.86880
FAM84B	58.40240	221.10499
FANCA	84.88066	39.61964
FANCD2	79.18439	33.51114
FANCG	40.87542	14.44347
FANCI	133.39284	66.45138
FEN1	131.38976	59.20110
FGF19	34.36540	87.00336
FLOT1	64.09867	27.45972
FNDC3B	58.15202	136.78481
FOSB	107.66574	300.74389
FOSL1	49.95189	203.46455
FOXM1	90.32655	40.59015
FRMD5	40.06167	110.69522
FSD1	30.23404	9.30548
FXYD5	39.56090	88.94438

GABBR1	64.28646	24.20566
GADD45A	41.06321	96.30883
GADD45B	9.95282	61.99846
GARS	245.37773	527.27232
GATA2	38.12118	7.42155
GDF15	70.60869	550.45038
GEN1	37.30743	12.04574
GFPT1	128.38513	271.28606
GINS1	71.48504	27.40263
GINS2	49.13814	15.47107
GLB1L2	59.02837	29.05821
GOT1	103.40919	237.66075
GPAT3	30.73481	75.07179
GPRC5A	236.86462	796.78862
GPT2	51.26642	123.08349
GRIN2B	102.21985	43.61586
GTPBP2	38.05859	590.58382
GTSE1	48.95035	20.38071
H2AFX	89.70058	26.37503
HBEGF	7.94974	46.41321
HELLS	135.64631	66.10885
HERPUD1	45.13197	375.75860
HES1	38.99753	160.81920
HJURP	59.21616	22.89262
HK2	64.72464	196.55680
HMGA2	192.92198	569.06133
HMGB2	211.13752	96.25175
HMGN2	229.72864	98.19277
HMMR	49.13814	20.89451
HOXB6	26.91643	5.93724
HOXB8	32.61270	2.45482
HS3ST1	56.83749	178.85926
HSP90AA1	3965.35416	1977.55664
HSP90B1	937.31788	2146.99625
HSPA13	50.89084	113.43548
HSPA5	889.36907	5169.22115
HSPH1	234.92414	113.15004
HTR7	17.15140	80.89485

HYOU1	291.63644	775.95121
ID2	103.40919	45.95650
ID3	117.99414	46.01359
IER3	303.34196	642.82008
IFRD1	31.61116	75.52851
IGFBP2	84.81807	38.53495
IL17RD	74.23928	31.45594
INCENP	85.50663	32.65480
IRF2BP2	39.62350	109.89597
IRS1	96.08541	374.44555
ITCH	113.80018	245.71027
ITPRIPL2	82.43940	169.15417
JDP2	11.45513	48.46841
JUN	62.53376	166.87061
KCNQ2	62.34597	27.34554
KDM6B	50.45267	122.68387
KIF11	83.75393	32.54063
KIF14	47.07246	15.98487
KIF20A	74.11409	23.92021
KIF20B	41.25100	11.64612
KIF22	85.69442	39.67673
KIF23	115.92846	49.38183
KIF4A	72.42399	33.39696
KIF5C	34.67838	11.64612
KIFC1	78.62103	29.51492
KLF4	25.03854	172.86494
KLF6	132.76688	550.67873
KNL1	74.05149	31.39885
KNTC1	112.73604	44.70054
KREMEN1	111.92229	50.52360
KRT23	37.11964	112.17953
KRT80	3.56799	31.85556
LAMB3	38.37157	132.67441
LAMC2	39.93648	88.08805
LDLR	85.88220	265.06338
LIF	52.01757	110.18142
LIG1	69.85754	20.83742
LINC00963	35.24175	91.17084

LMNB1	163.75208	69.19165
LSM4	147.79000	70.04798
LURAP1L	1.50231	32.36936
LY6E	97.96330	48.46841
MAFF	13.27043	69.02038
MALT1	76.93093	161.73262
MANF	59.15356	165.72884
MAP1A	24.28739	3.93913
MAP1B	70.60869	149.57270
MAPK12	63.22232	29.62910
MARCKS	81.87604	29.62910
MCM10	61.84520	28.71568
MCM2	135.70891	51.66538
MCM3	261.96576	129.76288
MCM4	332.69965	110.69522
MCM5	123.50262	49.78145
MCM6	196.99074	89.28691
MDC1	144.03422	68.10696
MEF2D	48.32439	107.15571
MELK	88.69904	39.50546
MIS18BP1	39.24792	14.27220
MKI67	908.39836	298.40325
MMP14	25.47672	4.11039
MRC2	40.68763	11.87447
MRPS34	65.35060	28.65859
MT2A	84.69287	29.97163
MTHFD1	190.16774	92.76933
MTHFD1L	91.82886	221.50461
MTHFD2	265.40856	662.11610
MTND2P28	19.27968	59.20110
MXD1	6.44742	33.68240
MYBL2	111.10854	38.30660
NBEAL1	37.99599	11.87447
NCAPD2	377.45605	162.07515
NCAPG	117.49337	49.61018
NCAPG2	116.99260	50.29525
NCAPH	78.37064	35.79469
ND2	5962.67878	15802.01339

ND3	1069.39620	2498.60627
NES	60.96885	21.00868
NFIB	23.84921	5.82306
NFIL3	30.29664	93.05477
NFKBIA	24.85075	66.10885
NGFR	34.42800	10.56143
NKD1	39.37311	14.44347
NR4A1	64.72464	248.05091
NR4A2	32.36232	247.65129
NR4A3	1.18933	44.41510
NRGN	21.22017	3.48242
NT5DC2	84.44249	26.48921
NT5E	29.92106	281.73331
OAS3	76.05458	36.99355
OCLN	24.78816	81.57992
OGFRL1	34.36540	93.22604
OR51B4	19.84305	55.31906
PALLD	243.12426	102.87405
PAM	545.65246	232.57984
PARD6B	21.65834	59.65781
PASK	46.00832	18.43969
PBX1	63.91088	22.43591
PCDH7	147.10144	344.58810
PCK2	26.10268	135.58594
PDIA4	405.56181	969.31103
PDK3	60.40549	26.77466
PDLIM5	526.05980	215.79573
PEG10	89.07462	5.99433
PHGDH	106.35121	296.23387
PHLDA1	437.54855	906.79878
PHLDA2	43.56707	96.13757
PKIB	15.58649	73.47331
PLAU	23.72402	179.77269
PLAUR	21.03238	85.00525
PLK1	97.21215	39.67673
PLK2	87.00894	229.15451
PMAIP1	46.94727	111.38028
PNRC1	38.05859	85.11943

POLA1	86.69596	38.30660
POLD1	53.20691	23.23515
POLE	221.09034	108.01204
POLQ	42.94110	16.78411
PPP1R15A	49.51372	211.28571
PRKACB	212.51464	445.46404
PRKDC	1416.74340	481.65835
PSAT1	133.33024	605.48400
PSPH	35.61733	79.86726
PTMAP5	85.81961	39.44837
PVR	56.33672	120.05778
PYGB	116.74221	235.14884
RACGAP1	146.91365	61.02795
RBCK1	39.06013	106.41356
RCN1	71.98581	157.05134
RECQL4	86.69596	30.99923
RFC2	58.77798	27.57390
RFC4	51.14123	21.52248
RGS2	20.53161	3.19697
RPE65	22.72248	72.16027
RPS4Y1	24.47518	0.05709
RPS6KA1	68.16744	31.62720
RRM1	178.46222	79.23928
RUNX1	39.68609	106.69900
S100A14	85.06845	237.14695
SAMD5	34.55319	7.53572
SAPCD2	56.14893	26.26086
SARS	132.76688	323.75068
SDC4	91.89146	379.86899
SEL1L	97.71292	275.91025
SEMA3A	28.60654	97.79314
SERINC1	79.55997	174.46343
SESN2	18.34073	91.11375
SESN3	38.80974	6.67939
SESTD1	70.73389	25.11908
SGO2	33.92723	7.07901
SH2B3	42.50293	102.18898
SH3KBP1	88.69904	205.29139

SHCBP1	53.14431	23.12097
SIPA1L2	22.59729	66.96518
SKP2	54.64662	23.74895
SLC2A4RG	49.45112	18.15424
SLC38A1	330.07060	707.44463
SLC38A2	1104.32497	2815.50630
SLC3A2	149.54270	484.28444
SLC5A1	0.81375	35.39507
SLC7A1	160.99784	390.37333
SLC7A11	57.27567	439.41263
SLC7A5	340.02342	1119.96842
SLFN5	51.39161	214.25433
SMAD3	100.34196	410.52569
SMC2	41.87696	15.35689
SMC4	133.89361	48.81094
SNTB1	6.57262	84.94816
SPAG5	75.42861	37.39318
SPCS1	49.07555	17.35500
SPTB	32.67530	9.07712
SRF	46.88467	101.16138
STARD4	32.55011	117.26043
STARD9	45.44496	12.21701
STC2	39.93648	190.79083
STK39	151.04501	336.70985
STMN3	80.06074	36.93646
SYNE2	212.57724	73.98711
TACC3	133.64323	54.00602
TACSTD2	2.37866	46.92701
TBX3	33.92723	9.87637
THBS1	18.77891	70.27633
THRA	47.82362	19.41020
TICRR	49.26333	20.09526
TIMELESS	134.70736	48.35423
TIMP3	16.83842	0.00000
TINAGL1	36.11810	81.52283
TK1	166.69410	46.69865
TMED7	97.21215	235.43428
TMEM41B	59.84212	164.41579

TMPO	336.07985	165.84301
TMSB4X	106.16343	235.20593
TNFRSF10B	140.15325	434.67426
TNFRSF10D	65.60098	141.92280
TNS4	91.76626	37.05064
TONSL	65.28800	22.20755
TOP2A	301.52666	103.95874
TPGS2	88.88683	42.13155
TPX2	338.02034	158.13602
TRIB3	18.96670	181.77079
TRIM38	52.14277	106.87027
TRIM8	45.69534	129.76288
TRNL1	19.90564	54.69109
TRNP1	51.82979	20.09526
TSC22D3	10.32840	39.16293
TSTA3	63.41011	29.40074
TTK	53.58248	19.75273
TUBA1B	293.26394	121.88463
TUBB4B	337.20659	161.90389
TXNRD1	436.60960	1103.01304
TYMS	61.53222	28.31605
UBE2J1	46.57169	144.09218
UPP1	7.26118	40.01926
VASH2	18.27814	0.11418
VASP	83.75393	168.69746
VCAN	148.29077	65.25252
VEGFA	50.20228	168.46910
VPS13A	180.21492	66.45138
WARS	77.74468	309.87810
WDR34	50.26488	22.72135
WNT16	58.90317	235.54846
XBP1	59.15356	137.46987
YARS	189.54178	454.31281
YBX1	1128.86274	350.92496
YIPF6	60.65587	122.17007
ZFP36	53.70768	124.51071
ZGRF1	30.42183	7.70699
ZMAT3	60.15510	122.74096

ZWINT	62.97194	19.01058
